# Activation dynamics of a water-soluble human mu-opioid receptor

**DOI:** 10.1016/j.jbc.2026.111393

**Published:** 2026-03-20

**Authors:** Eugene Agyemang, Raegan Van Wirt, Calixte Walls, Thomas T. Joseph, Sriram Tiruvadi-Krishnan, John Grothusen, Qiu Zhang, Alan Hicks, Wellington Leite, Naresh C. Osti, Eugene Mamontov, Hugh M. O’Neill, Renyu Liu, Rajan Lamichhane

**Affiliations:** 1 UT-ORII Genome Science and Technology Graduate Program, University of Tennessee, Knoxville, Tennessee, USA; 2 Department of Biochemistry & Cellular and Molecular Biology, University of Tennessee, Knoxville, Tennessee, USA; 3 Department of Chemistry, University of Tennessee, Knoxville, Tennessee, USA; 4 Department of Anesthesiology and Critical Care, University of Pennsylvania Perelman School of Medicine, Philadelphia, Pennsylvania, USA; 5 Neutron Scattering Division, Oak Ridge National Laboratory, Oak Ridge, Tennessee, USA

**Keywords:** allosteric modulation, g protein, G protein-coupled receptor (GPCR), mu-opioid receptor, neutron scattering, single-molecule FRET (smFRET), structural dynamics

## Abstract

The mu-opioid receptor (MOR), a class A G protein-coupled receptor mediates opioid analgesia and remains a central target for pain therapeutics. While crystal structures of MOR exist, they provide limited insight into the receptor’s dynamic conformational landscape underlying function. Here, we engineered a thermostable water-soluble MOR variant (wsMOR) that retains native-like ligand-binding and activation dynamics. This variant enables high-yield production and detailed solution-phase structural studies that are challenging with membrane-embedded MOR, providing a valuable tool for studying receptor activation and aqueous-phase drug screening. Using a combined computational and experimental approach, we performed long-timescale all-atom molecular dynamics simulations together with neutron scattering and single-molecule FRET, revealing a structurally stable receptor with a diverse ensemble of conformations at different temporal resolutions. In the ligand-free state, wsMOR displayed high conformational flexibility, which decreased upon agonist binding, particularly in transmembrane helix 6, a hallmark of G protein-coupled receptor activation. Positive allosteric modulation and G protein binding further stabilized active-like states. These findings highlight wsMOR’s conformational plasticity across picosecond to millisecond timescales and provide a foundation for structure-guided development of next-generation opioid ligands with improved efficacy and safety.

The involvement of G Protein-Coupled Receptors (GPCRs) in many human physiological processes has made them important targets for pharmaceutical drug development, with 36% of currently approved drugs targeting these receptors (1). Despite their importance in human health, studying receptor-ligand interactions and conformational dynamics at the molecular level remains challenging due to technical limitations. In recent years, significant advancements in the structural studies of GPCRs have provided valuable insights into receptor-ligand complex interactions (2, 3, 4, 5, 6). However, information on the conformational dynamics of GPCRs during ligand recognition and receptor activation is still lacking, leaving a critical knowledge gap that limits our ability to design and develop effective drugs with minimal side effects.

Membrane proteins such as GPCRs typically require lipid environments or membrane mimetics for stability (7), limiting both yield and suitability of dynamic measurements. Recent efforts to engineer soluble variants of druggable integral membrane proteins, including GPCRs, have used experimental and computational design strategies to enable high-yield purification while preserving native-like α-helical structure and ligand-binding ability, providing new tools to investigate receptor dynamics and function (8, 9, 10, 11, 12). However, for such soluble GPCR variants to be broadly useful, it is essential that they preserve not only structure and ligand binding, but also the conformational dynamics and functional transitions that underlie receptor signaling.

The mu-opioid receptor (MOR), a class A GPCR, is the primary molecular target for clinically and recreationally used opioids like morphine and heroin (13). MOR activation mediates analgesia but also leads to undesired side effects, including respiratory depression, constipation, and opioid addiction (13, 14). Previous structural and biophysical studies have defined the inactive and active states of MOR and outlined the major conformational rearrangements associated with receptor activation, particularly the outward displacement of transmembrane helix 6 (TM6), which enables G protein or β-arrestin coupling (2, 6, 15, 16, 17, 18, 19). However, these techniques often provide static snapshots or ensemble averages, leaving transient conformational transitions and ligand-specific dynamics poorly characterized. Recent work on mouse MOR has suggested the existence of intermediate or pre-activated conformations that are difficult to resolve with traditional structural approaches and require methods capable of capturing conformational transitions in solution (20).

Here, we use several biophysical techniques, including neutron scattering and single-molecule Förster resonance energy transfer (smFRET), to investigate a soluble, thermostable human MOR variant (wsMOR) engineered for detergent-minimal purification while maintaining native-like structure and ligand-binding (8, 9). Using this variant, we show that ligand binding and G protein coupling modulate the conformational dynamics of the intracellular half of TM6, revealing intermediate and fully active conformations. While related conformational states have been observed in micelle-reconstituted or truncated MOR constructs (20, 21, 22), this work demonstrates ligand- and G-protein-dependent conformational dynamics using a soluble GPCR variant. This framework offers a solution-based alternative to traditional membrane-based approaches and a path toward extending these methods to poorly characterized GPCRs relevant to drug discovery.

## Results

### Soluble human MOR from *Escherichia. coli* for structural studies

**Figure 1:**
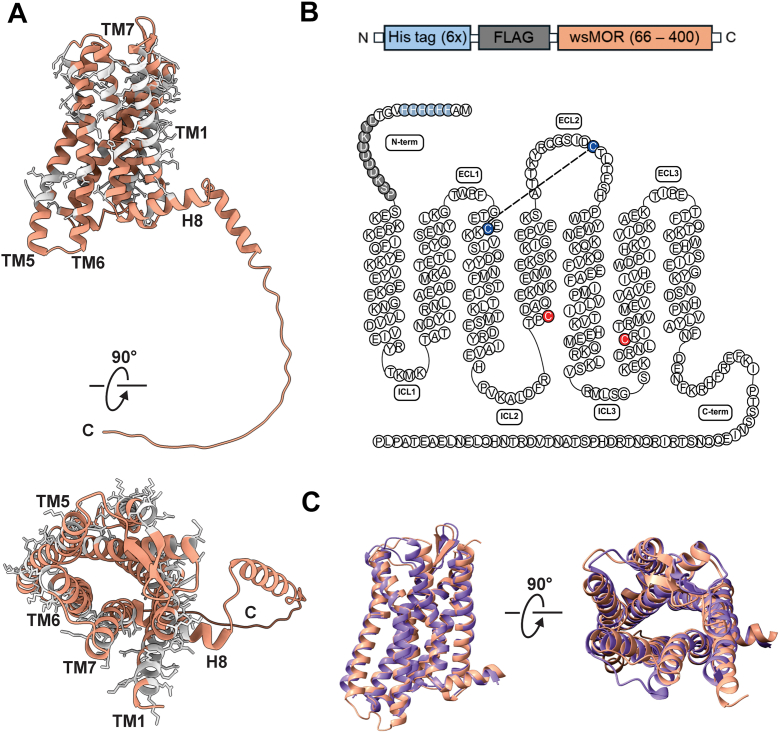
**Structural characterization of wsMOR.***A*, AlphaFold3 predicted structure of human wsMOR (*brown*) with mutated residues on the transmembrane regions highlighted in *gray* [see Perez-Aguilar *et al.* (2013) for details]. *B*, wsMOR construct used for all structural and functional studies and secondary structure diagram illustrating the modified primary sequence of the minimal cysteine wsMOR construct. Cysteines in TM3 and ECL2 involved in disulfide bond formation are shown in *blue*. Nonnative cysteines (Cys184 and Cys278) introduced for site-specific fluorophore conjugation are highlighted in *red*. The wsMOR construct is N-terminally truncated, starting at residue 65. *C*, superimposition of the lateral (*left*) and extracellular (*right*) views of antagonist-bound mouse MOR (*purple*; PDB: 4DKL) and unliganded wsMOR (*brown*) with RMSD of 1.08 Å. The AlphaFold3 model is shown for qualitative comparison of overall receptor topology. The C-terminal tail of wsMOR was removed for visual clarity. ECL, Extracellular loop; ICL, Intracellular loop; N-term, N-terminus; C-term, C-terminus; TM, Transmembrane; wsMOR, water-soluble MOR.

To facilitate structural and functional characterization of human MOR independent of membrane-specific interactions, we expressed a computationally engineered, water-soluble variant of the receptor in *Escherichia. coli* (8). In this variant, 53 hydrophobic amino acid residues (∼18% of the receptor) on the transmembrane surface were redesigned using homology modeling to enhance solubility and expression yield (Fig. 1*A*) (8, 9). Additionally, 11 cysteine residues were mutated to polar or charged amino acids, with two cysteine residues retained for disulfide bond formation (Fig. 1*B*) (8, 9). Cysteine mutations were introduced to reduce non-specific disulfide bond formation and generate a minimal cysteine platform for site-specific labeling in single-molecule fluorescence microscopy. The selected cysteines were predicted to be solvent-exposed and not involved in conserved structural or ligand-interacting regions, thereby minimizing potential effects on receptor structure and function. For our study, the receptor construct was truncated at the N-terminus and had hexahistidine and FLAG tags attached to the N-terminus to aid in purification and detection (Fig. 1*B*). Purification using Ni^2+^-NTA affinity chromatography, followed by SDS-PAGE analysis (Fig. S1*A*), revealed that the receptor yield was ∼21 mg/L of *E. coli* culture and was present as monomers and dimers. We next generated an AlphaFold3-predicted structure of wsMOR (Fig. S1*B*), which showed a high degree of structural similarity to the antagonist-bound mouse MOR (PDB: 4DKL), with a RMSD of 1.08 Å (Fig. 1*C*), indicating that the engineered receptor retains its native-like overall topology. Notably, the water-soluble variant exhibits a markedly reduced hydrophobic surface compared to the native MOR (Fig. S1*C*).

### Conformational stability of unliganded wsMOR

**Figure 2:**
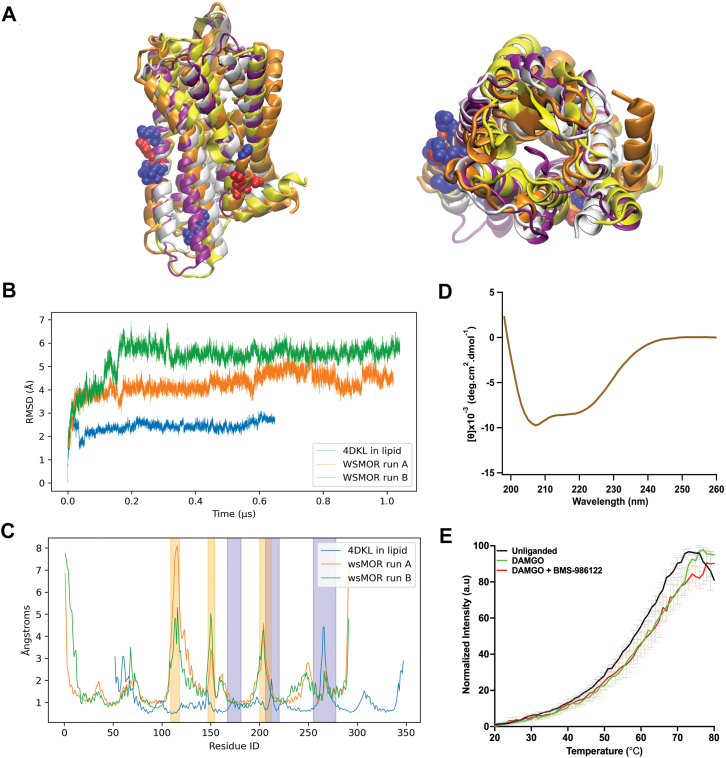
**Structural stability and thermal unfolding of wsMOR.***A*, all-atom MD simulation of wsMOR homology model, side view (*left*) and top view (*right*). The initial structure (*silver*) and final structure (run A, *yellow*; run B, *orange*) are shown after 1 μs of simulation. Mouse MOR after 649 ns of simulation is shown in *purple*. Residue side chains that differ between the homology model and AlphaFold3 model are depicted in sphere representation and colored by charge (*blue: positive; red: negative; white: neutral*). Note the rotation of extracellular regions of nominal transmembrane domains. *B*, RMSD of the simulated structure with respect to the initial homology model. *C*, root-mean-square fluctuation of the simulated structures with respect to the initial homology models, as a function of residue ID. Intra and extracellular loops are highlighted in orange shading for the wsMOR simulations, and *blue* for mouse MOR. Note the greater fluctuations in the highlighted regions. *D*, far UV CD spectrum of wsMOR (0.3 mg/ml) recorded at RT, displaying features characteristic of a α-helix. The α-helix contents were calculated by the server BestSel. *E*, thermal fluorescence unfolding curves of wsMOR monitored by N-[4-(7-diethylamino-4-methyl-3-coumarinyl)phenyl]maleimide fluorescence. The unfolding curves for the unliganded state (*black*, Tm = 55.0 °C), DAMGO-bound state (*green*, Tm = 59.7 °C), and DAMGO + BMS-986122-bound state (*red*, Tm = 59.5 °C) are shown. Data are presented as mean ± SEM (n = 3). DAMGO, [D-Ala^2^, *N*-MePhe^4^, Gly-ol]-enkephalin; wsMOR, water-soluble MOR.

We previously generated homology models of wsMOR, in which some hydrophobic residues at the lipid-facing surfaces of the TM domain were substituted with more hydrophilic residues without altering their opioid-binding capabilities or basic structural features (8). To determine whether the wsMOR variant used in this study is stable in an aqueous environment, we conducted two independent all-atom MD simulations of the unliganded wsMOR (without any bound ligand) homology model, each (“run A “and“run B”) 1 μs in length (Fig. 2, *A* and *B*). The RMSD of the protein alpha carbons relative to the initial homology model varied from 1.4 to 5.9 Å over the course of run A (mean 4.2 ± 0.5 Å) and from 1.5 to 7.0 Å over the course of run B, with a mean of 5.4 Å ± 0.7 Å (Fig. 2*B*). Since the homology model was built from a transmembrane template but designed to be soluble in a profoundly different environment lacking the stabilizing influence of a lipid membrane, the model was expected to relax during simulation, consistent with the relatively large RMSD. After excluding the first 200 ns of the respective simulation runs to account for equilibration of wsMOR, the RMSD ranges were 3.5 to 5.9 Å and 4.7 to 6.9 Å, respectively. Throughout the simulation, no significant loss of secondary helical structure was observed, and the overall TM helical fold remained preserved (Fig. 2, *A*–*C*).

As a comparison, we conducted a 649 ns simulation of mouse MOR (PDB: 4DKL) embedded in a 70:30 mol% (1-palmitoyl-2-oleoyl-sn-glycero-3-phosphocholine): cholesterol lipid bilayer. This system exhibited an RMSD range of 0.0 to 3.1 Å relative to the initial 4DKL structure after MD minimization and equilibration in the lipid bilayer, with a mean of 2.4 Å ± 0.2 Å. After omitting the first 200 ns of production simulation, the RMSD range was 2.0 to 3.1 Å (mean 2.5 Å ± 0.2 Å), consistent with lipid membrane-mediated stabilization of the receptor structure (Fig. 2*B*). A per-residue root-mean-square fluctuation analysis showed that in both wsMOR and mouse MOR, intra- and extracellular loop regions fluctuated the most, whereas the TM helices exhibited substantially lower flexibility (Fig. 2*C*). These data indicate that, even without the stabilizing influence of a lipid bilayer, the substitution of hydrophilic for lipophilic side chains in the transmembrane domain yields a wsMOR variant that retains its overall helical fold and conformation in the aqueous phase.

We next sought to experimentally validate the folding and structural integrity of purified wsMOR. To access the receptor’s secondary structure, we first acquired CD spectra. The analysis revealed that the receptor variant exhibits an alpha-helical protein fold with a helical content of ∼47.6%, typical of GPCRs with seven predicted transmembrane domains (Fig. 2*D*), This value is consistent with the 40 to 60% α-helical content reported for native class A GPCRs in detergent micelles (23, 24, 25) and closely matches the ∼50% helical content measured for full-length human MOR in SDS micelles (26), which displays similar characteristic CD minima at 208 nm and 222 nm. Furthermore, ligand binding is a hallmark of functional GPCRs, stabilizing receptor conformations critical for signaling. For this purpose, we conducted fluorescence thermal shift assays using a thiol-reactive dye, N-[4-(7-diethylamino-4-methyl-3-coumarinyl)phenyl]maleimide (CPM), which reacts with cysteines that become exposed due to thermal denaturation (27). Thermal unfolding curves obtained from the assay were typical of well-folded proteins (Fig. 2*E*). For the unliganded wsMOR, the thermal unfolding curve indicated that it was relatively stable, with a melting temperature of ∼55.0 °C. The addition of saturating concentrations of the synthetic high-efficacy agonist [D-Ala^2^, *N*-MePhe^4^, Gly-ol]-enkephalin (DAMGO) shifted the thermal spectrum by ∼5 °C, increasing the melting temperature to ∼59.7 °C, indicating ligand binding. Similarly, saturating concentrations of the positive allosteric modulator (PAM) BMS-986122 caused a right shift to ∼59.5 °C (Fig. 2*E*). As a control, thermal unfolding spectra were measured in the presence of glucagon, a class B glucagon receptor agonist that does not interact with the class A MOR - and showed no significant changes in melting temperature (Fig. S2). The increase in melting temperature with agonists is consistent with reports that ligand binding enhances GPCR stability (28, 29). Collectively, these findings indicate that the engineered MOR maintains structural and thermal stability, exhibits enhanced hydrophilicity relative to the native receptor, and retains its ligand-binding capability.

### wsMOR exists as monomers and dispersed oligomers in solution

We used small-angle X-ray and neutron scattering (SAXS/SANS), and analytical ultracentrifugation (AUC) to determine the solution structure and oligomeric state of wsMOR in its unliganded form. Both SAXS and SANS provide complementary information on the size and overall shape of proteins in solution (30, 31). The SAXS data for wsMOR at 0.7 mg/ml and 2.76 mg/ml (both in 0.01% SDS) yielded radius of gyration (*R*_*g*_) values of 43.4 ± 0.2 Å and 59.8 ± 0.9 Å (Fig. S3 and Table S1), respectively, indicating a concentration-dependent increase in apparent particle size. AUC experiments at 0.5 mg/ml, 2.5 mg/ml, and 10 mg/ml (Fig. S4 and Table S2) further supported the SAXS results, confirming that wsMOR forms large particles in a concentration-dependent manner in solution. Analysis of the AUC data indicated monomers as the predominant species, with their distribution varying by receptor concentration. In addition, species with sedimentation coefficients consistent with dimers, trimers, and tetramers were detected, although these species were much less abundant (Fig. S4 and Table S2).

**Figure 3:**
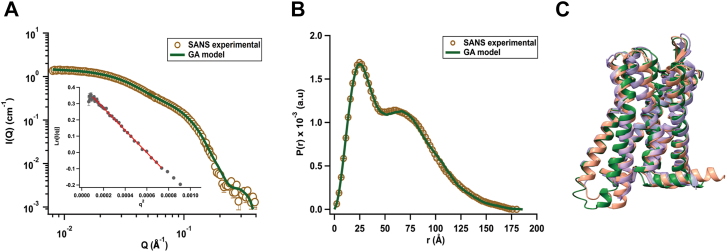
**Solution properties of unliganded wsMOR.***A*, experimental SANS scattering curve (*brown circles*) of unliganded wsMOR overlaid with the theoretical SANS curve calculated from a GA-refined ensemble of monomers and oligomers. The inset shows the Guinier plot for the q∗Rg < 1.3 region of the scattering curve. *B*, pair-distance distribution function P(r) calculated from the SANS data (*brown circles*) shown in *A*. The theoretical P(r) profile calculated from the GA-refined ensemble of monomers, dimers, trimers, and tetramers is shown as the green fit line. *C*, superimposition of AlphaFold3-predicted human wsMOR (*brown*), GA monomer model (*green*), and antagonist-bound mouse MOR (*purple*; PDB:4DKL). The AlphaFold3 and BilboMD/GA models are shown for qualitative comparison of overall topology. Parameters derived from SANS analysis are presented in Table S1. In *Panel A*, error bars for the SANS data are represented by solid vertical lines and are derived from counting statistics errors (N1/2/N), where N represents the number of detector counts. For the inset, errors were propagated from the SANS data. In *panel B*, the errors for the P(r) analysis are shown as solid vertical lines and represent standard deviations based on multiple fits to the data using a series of Monte Carlo simulations [Svergun and Pederson (1994)]. wsMOR, water-soluble MOR.

Next, we used SANS with isotopic labeling, which allowed us to study the solution structure of MOR without detergent contributions (Fig. 3*A*). We prepared protiated wsMOR at 2.5 mg/ml with deuterated SDS (d-SDS, 0.01%) in a 98% D_2_O buffer. Under these conditions, the scattering signal of d-SDS was selectively masked, as its neutron scattering length density matched that of the deuterated buffer, highlighting the signal of the protiated receptor. This approach allowed us to uniquely isolate the receptor’s scattering features from the detergent environment (30, 31). Based on SAXS and AUC results (Fig. S3; Tables S1 and S2), which initially suggested a polydisperse oligomeric population of wsMOR in solution, we generated full-length AlphaFold3 models for the main species observed (monomers, dimers, trimers, and tetramers). All AlphaFold3-predicted oligomeric assemblies exhibited low interface confidence (ipTM < 0.5), indicating uncertain protein-protein interactions (Figs. S5–S7). These models were used as structural templates and were subjected to fast molecular dynamics ensemble sampling using BilboMD in combination with a Genetic Algorithm (GA) to identify the best models that fit the SANS data. Flexible regions within wsMOR were identified and defined using AlphaFold3’s Predicted Aligned Error (PAE).

The GA explored various combinations of AlphaFold3-BilboMD models, simultaneously searching for the optimal mixtures of monomers, dimers, trimers, and tetramers that best fit the experimental data. The analysis revealed that the minimal ensemble required to best fit the data was a four-state ensemble consisting of monomers (82.1%) and tetramer-like species (10.5%), with smaller contributions from dimers (3.7%) and trimers (3.7%), yielding a χ^2^ of 1.92. Notably, the GA-selected dimer- (Fig. S5) and tetramer-like (Fig. S7) states exhibited large inter-subunit separations of ∼30 Å. Such distances do not correlate with stable protein–protein interfaces and instead indicate that these apparent oligomeric states arise from detergent-mediated organization, in which SDS likely forms micelle-like shells around individual receptors (32), contributing to their apparent size in SAXS and AUC. Interestingly, the trimer state selected by the GA forms a compact assembly without the large separations observed in the dimer- and tetramer-like species and closely resembles the AlphaFold3-predicted trimer (Fig. S6). However, the predicted interaction interfaces in the AlphFold3 trimer are of low confidence (Fig. S6), indicating weak or transient inter-subunit contacts, and therefore cannot be interpreted as a stable oligomer.

Additionally, the theoretical *P(r)* profile of the four-state ensemble closely overlays very well with the experimental SANS *P(r)* profile, although the theoretical *P(r)* has a slight secondary peak at 74 Å. The experimental P(r) profile exhibits a peak around 25 Å and a broad shoulder between 50 Å and 80 Å (Fig. 3*B*), corresponding to molecular density distributions within the monomeric receptor and those spatially separated structures, respectively. The broadening of the 50 Å to 80 Å shoulder is likely due to polydispersity in the distribution of center-to-center distances among the monomers in the micelle-like state. Consistent with the agreement in the *P(r)* profiles, the R_*g*_^GNOM^ (47.8 ± 1.3 Å) of the MD-derived four-state ensemble is identical to the experimental R_*g*_^GNOM^ (47.7 ± 0.3 Å) and R_*g*_^Guinier^ (47.6 ± 1.9 Å) values derived from the SANS data (Table S1), indicating that the MD-derived ensemble captures the overall solution organization and size of wsMOR observed experimentally. For comparison, the conformation of the wsMOR monomer predicted by AlphaFold3 closely resembles the crystal structure of the mouse MOR bound to the antagonist β-funaltrexamine (β-FNA) (PDB: 4DKL [66–350]), with an RMSD of 1.08 Å (Fig. 3*C*). The Bilbo-MD/GA monomer also aligns well with the crystal structure (PDB: 4DKL), showing an RMSD of 1.15 Å, while the AlphaFold3 model exhibits an RMSD of 0.93 Å when superposed onto the Bilbo-MD/GA model (Fig. 3*C*). Together, the SAXS and AUC results suggest concentration-dependent apparent oligomerization of wsMOR, while contrast-matched SANS combined with the GA-based ensemble modeling demonstrates that wsMOR is predominantly monomeric and that the observed higher-order species arise from SDS-mediated, micelle-like organization rather than stable protein-protein interfaces.

### wsMOR internal dynamics indicate reduced flexibility upon ligand binding

**Figure 4:**
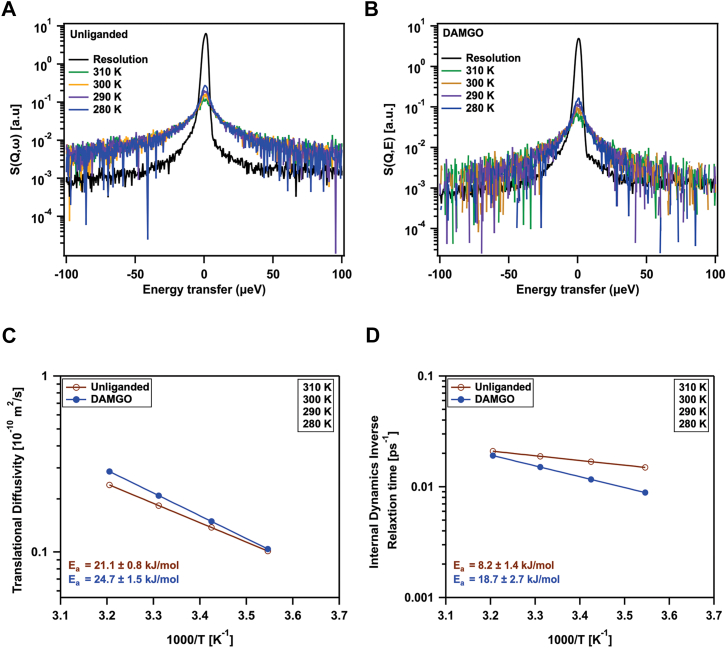
**Ligand binding slows wsMOR’s internal dynamics.***A*, quasi-elastic neutron scattering spectra of unliganded wsMOR (*B*) and DAMGO-bound wsMOR at Q = 1.5 Å^−1^, measured across different temperatures. The black spectrum represents the experimental resolution function, measured at 15 K. *C*, arrhenius plot showing the global diffusion of wsMOR, with or without DAMGO, measured by quasi-elastic neutron scattering. *D*, arrhenius plot depicting the temperature dependence of the rate of internal dynamics of wsMOR with or without DAMGO. DAMGO, [D-Ala^2^, *N*-MePhe^4^, Gly-ol]-enkephalin; wsMOR, water-soluble MOR.

To probe the dynamic properties of the receptor, we employed quasi-elastic neutron scattering (QENS), a technique that allows for the quantification of both global diffusivity and rates of internal motion on a neutron spectrometer such as BASIS (33, 34). The QENS spectra for both the unliganded and DAMGO-bound forms of wsMOR were measured across a temperature range of 280 K to 310 K and analyzed over a Q range of 0.7 to 1.5 Å^−1^. Figure 4, *A* and *B* shows representative QENS spectra of unliganded and DAMGO-bound wsMOR at the largest scattering vector Q = 1.5 Å^−1^. The spectra were fitted with a sum of two Lorentz functions (convolved with the spectrometer’s resolution function) and a linear background term. From the slope of the Q^2^-dependence of the slower dynamic component (Fig. S8*A*) (the narrower Lorentz function, for which the half-width at half maximum, HWHM = DQ^2^, where D is the diffusivity), we generated an Arrhenius plot for the global (predominantly translational) diffusivity of the receptor (Fig. 4*C*). We observed that both the unliganded (with an activation energy of 21.1 ± 0.8 kJ/mol) and DAMGO-bound (with an activation energy of 24.7 ± 1.5 kJ/mol) states of the receptor exhibited similar temperature dependencies (Fig. 4*C*), even though the ligand-bound state showed a slightly higher activation energy. This similarity in the activation energies suggests that the energy barrier for the global molecular motion (diffusion) remains largely unchanged by ligand binding. In the context of protein-ligand interactions, our findings imply that the hydrodynamic properties of the receptor do not change significantly upon ligand binding in solution.

To determine the influence of the ligand DAMGO on the internal dynamics of the receptor during activation, we examined the molecular motions of wsMOR by analyzing the Arrhenius plot for its internal dynamics. Using power law fits of the Q-dependence of the broader Lorentz component (internal dynamics), HWHM = (1/τ_0_)Q^α^, we found that the internal motions, quantified by the inverse relaxation time (the rate), (1/τ_0_), were faster than those observed for the global translational diffusion (Figs. 4 and S8*B*). As the temperature decreased, the internal dynamics slowed down, with a notable difference observed in the presence of the ligand (Fig. 4*D*). Specifically, the activation energy for the ligand-bound receptor was much higher (18.7 ± 2.7) kJ/mol compared to the unliganded state (8.2 ± 1.4) kJ/mol. At the physiological temperature of 310 K (37 °C), the internal dynamics of the ligand-bound receptor were only slightly slower compared to the unliganded state (Fig. 4*D*). Yet, the large difference in the activation energy indicates the much-increased energy barriers between the internal states as induced by ligand binding.

**Figure 5:**
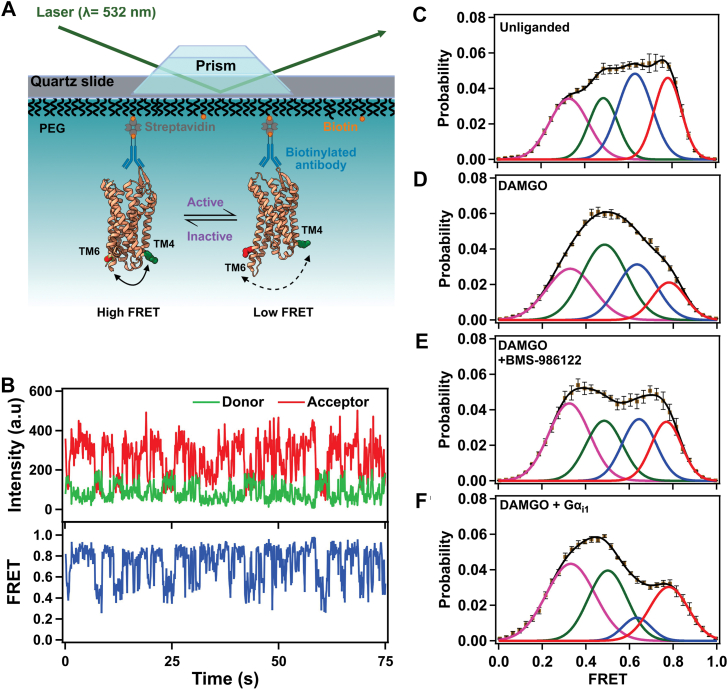
**Ligand binding and G protein interaction modulate transmembrane helix 6 dynamics in wsMOR.***A*, schematic of the single-molecule TIRF microscopy setup, illustrating immobilization of fluorophore-labeled wsMOR *via* a biotin-PEG/streptavidin/biotinylated anti-FLAG antibody sandwich on a quartz microscope slide. *B*, representative single-molecule fluorescence time trace of unliganded wsMOR showing donor (*green*) and acceptor (*red*) intensities, along with the corresponding FRET efficiency time trace (*blue*), calculated till the photobleaching point, from the intensities (I) according to the equation: I(LD655)/[I(LD555) + I(LD655)]. The anti-correlated fluctuations in donor and acceptor intensities are indicative of conformational dynamics consistent with the FRET states identified by step transition and state identification population analysis. *C* and *F*, FRET efficiency population histograms of wsMOR under different conditions: (*C*) unliganded, (*D*) DAMGO-bound, (*E*) DAMGO + BMS-986122-bound, and (*F*) DAMGO + GDP-bound Gαi1. Histograms in *panels C*–*F* were generated from 370, 154, 82, and 94 molecules, respectively. Cumulative Gaussian fits are shown in *black*. *Magenta*, *green, blue*, and *red lines* represent low (0.33), intermediate (0.49), intermediate (0.63), and high (0.78) single-molecule Förster resonance energy transfer efficiency states, respectively. The distinct FRET efficiency states were assigned based on results from step transition and state identification analysis. Error bars in *panels C*–*F* represent the standard error for each histogram bin. DAMGO, [D-Ala^2^, *N*-MePhe^4^, Gly-ol]-enkephalin; wsMOR, water-soluble MOR.

While QENS probes internal protein dynamics on much shorter time and length scales compared to global conformational dynamics, these local internal motions serve as essential precursors to global structural changes, as described by the protein energy landscape framework (35). The energy barriers associated with these local fluctuations ultimately determine how efficiently the conformational landscape can be explored and how readily distinct conformational states can be reached. Although the structural fluctuations measured by QENS at 310 K decrease only slightly upon DAMGO binding (55 ± 4) ps vs. (47 ± 2) ps, the marked increase in the activation energy (potential barriers) predicts an overall reduction in protein flexibility in the ligand-bound state. This interpretation is supported by smFRET (Fig. 5), which shows that DAMGO drives the receptor to populate distinct open (active) conformations while significantly reducing transitions to inactive or intermediate states. Thus, although the receptor adopts an open conformation, it is dynamically less flexible because it is effectively stabilized in this state. These effects are consistent with DAMGO binding suppressing fast, local side-chain and loop motions that are thought to facilitate the exploration of inactive and intermediate conformational states of the receptor. Our observations also align with previous QENS studies of Cytochrome P450, where substrate (camphor) binding reduced structural flexibility (36). Together, these findings suggest that agonist binding suppresses the receptor’s intrinsic molecular motions, altering its conformational energy landscape and stabilizing functionally relevant states.

### TM6 conformational dynamics reflect activation of wsMOR

To determine the activation process of wsMOR in real-time at the structural level, we used total internal reflection fluorescence (TIRF)-based smFRET microscopy. This technique allowed us to monitor the conformational dynamics of the receptor by observing the outward displacement of TM6 relative to TM4 in response to ligand binding. The receptor was site-specifically labeled with two fluorophores introduced at positions C184 and C278 (Fig. 5*A*) on the intracellular side of TM4 and TM6, respectively, allowing us to probe the distance change between the fluorophores during receptor activation. For single-molecule FRET imaging, sample immobilization was facilitated by a sandwich of interactions between biotinylated-PEG, streptavidin, and a biotinylated anti-FLAG tag antibody bound to the FLAG-tagged receptors (Fig. 5*A*). Control experiments confirmed the specific immobilization of the receptors on the microscope slide (Fig. S9). Labeled samples were then illuminated with a 532 nm laser, and data were acquired with exposure times of 100 ms. FRET efficiencies were subsequently calculated based on background-corrected donor and acceptor fluorescence intensities.

Single-molecule fluorescence time trajectories (Figs. 5*B* and S10) revealed that the receptor adopts four distinct conformational states defined by their FRET efficiencies: a high-FRET state (0.78), two intermediate-FRET states (0.63 and 0.49), and a low-FRET state (0.33) (Fig. 5*B*). These states were determined by applying step transition and state identification (STaSI) analysis to the averaged FRET histogram (37). STaSI identifies stepwise transitions in the FRET traces and selects the optimal number of states using a minimum description length criterion, providing a quantitative foundation for the four-state assignment. The distribution of these four states in the population histograms (Fig. 5, *C*–*F*) indicates that their transitions are slower than the 100-ms measurement window, and the observed width of each fitted peak is consistent with fast, time-averaged dynamics occurring within each conformational state. The relative proportions of these four states across all tested conditions are summarized in Figure S10. In the unliganded condition, the population was broadly distributed across the four states, with the ensemble dominated by the intermediate-FRET state at 0.63 (32.7%) and the high-FRET state at 0.78 (24.3%) (Fig. 5*C*).

Although the ensemble shows roughly equal populations across states, individual molecules sample only a subset of these conformations during the observation period (Fig. S10), reflecting slow interconversion between states (38). When compared with recent smFRET studies of mouse MOR in detergent micelles, the intermediate FRET state at 0.49 observed here closely corresponds to the active-like low-FRET state(∼0.5) resolved by Zhao *et al.* in the presence of Gi (20), providing a direct comparison between the two systems. The four-state ensemble resolved here likely reflects the broader conformational sampling accessible in the absence of native-like environments and the sensitivity of STaSI analysis in resolving distinct subpopulations. The relative proportions of these four states across all tested conditions are summarized in Figure S11. Upon ligand addition, the distribution shifted towards active-like conformations. DAMGO increased the population of the active-like states, with a marked rise in the 0.49 FRET state (from 19.5% to 34.9%) and a corresponding decrease in the high-FRET inactive state (Fig. 5*D*). Addition of BMS-986122 further enhanced this effect, markedly increasing the low-FRET population (0.33; from 26.7% to 34.4%) while reducing the intermediate FRET populations (0.49 and 0.63) (Fig. 5*E*). Together, these findings demonstrate that orthosteric and allosteric ligands cooperatively modulate wsMOR’s conformational dynamics.

To quantitatively interpret the observed FRET changes, we calculated the corresponding inter-fluorophore distances. We first determined the Förster radius (R_0_) for the LD555 (donor)–LD655 (acceptor) FRET pair to be ∼54.8 Å (Fig. S12). This value was calculated based on the spectral overlap integral between the donor emission and acceptor absorption spectra, the donor quantum yield, the refractive index of the medium (n = 1.4), and the orientation factor κ^2^ = two-thirds (39). We then compared our FRET-derived distances to distances estimated from available structural models of MOR (Table S3). In the inactive state, TM6 adopts an inward position, whereas in DAMGO-bound MOR-Gi complexes, TM6 is displaced outward by ∼10 Å. The smFRET-derived distances reflect a broader dynamic range: the intermediate-FRET state at 0.49, with a displacement of ∼10.8 Å relative to TM4, closely matches the ∼10 Å outward movement observed in static, active MOR structures (15, 40), supporting its assignment as an active-like conformation consistent with Zhao *et al.* (20), supporting its assignment as an active-like conformation. The low-FRET state at 0.33, with a larger displacement of ∼17.3 Å, represents a more open conformation than those captured in static structures. The intermediate-FRET state at 0.63, with a smaller displacement of ∼5 Å, likely represents an intermediate inactive state, distinct from the fully inactive or active-like conformations. The existence of these three distinct populations beyond the inactive state, and the fact that they are modulated by different ligands (Fig. S11), provides further evidence that the receptor exists as a conformational ensemble and that different ligands stabilize different subsets of these conformations (17, 20, 21). Based on the estimated distance changes and their comparison to resolved structures, we assigned the high-FRET state (0.78) to the inactive conformation, the intermediate-FRET state at 0.63 to the intermediate-inactive state, and the intermediate-FRET (0.49) and low-FRET (0.33) states to the active-like and active conformations of wsMOR, respectively.

### G protein coupling promotes conformational changes associated with wsMOR activation

GPCR activation is driven by ligand-induced conformational changes that facilitate G protein coupling and downstream signaling (41). Previous studies have shown that the inhibitory Gα_i1_ subunit binds to wsMOR with high affinity in the presence of the native human MOR C-terminal tail (42). Furthermore, recent work on the mouse MOR has revealed that G protein interactions stabilize distinct receptor conformations, including pre-activated and fully activated states, depending on the bound ligand (20). To probe these dynamics, we used smFRET to investigate DAMGO-bound wsMOR in complex with inactive GDP-bound Gα_i1_. smFRET analysis revealed a redistribution of FRET populations upon Gα_i1_ binding. The low-FRET state (0.33) increased markedly (40.7% *versus* 26.7% with DAMGO alone), consistent with the outward displacement of TM6 (Figs. 5*F* and S11). This shift was accompanied by a significant decrease in the intermediate-inactive 0.63 FRET state (7.1% *versus* 24.6%), while the intermediate-active state (0.49, 29.5%) remained populated. Unexpectedly, the high-FRET state (0.78) also showed a notable increase in relative population (22.3% *versus* 13.9%), which likely reflects a subpopulation of non-functional receptors not engaged with the G protein. These observations indicate that G protein coupling favors the low-FRET, active conformation, while reducing both the intermediate-inactive and intermediate-active states stabilized by DAMGO alone (Figs. 5*F* and S11). Similar observations have been reported for other class A GPCRs, such as β_2_AR and mouse MOR. (17, 43). Furthermore, the transition to the low-FRET state implies a more open receptor conformation that facilitates full Gα_i1_ binding, a key step in priming G proteins for nucleotide exchange and subsequent signal transduction. This conformation corresponds to an additional ∼7 Å displacement of the intracellular half of TM6 relative to TM4, a displacement not observed in available structures. This extra displacement may be a consequence of the absence of a lipid membrane or detergent micelles surrounding the receptor. These findings are consistent with the mechanism of G protein activation and align with previous reports that G protein interaction modulates GPCR conformational ensembles to facilitate activation (17, 20, 44).

## Discussion

In this work, we employed several complementary biophysical approaches, including neutron scattering and smFRET, to demonstrate the effectiveness of a thermostable water-soluble MOR construct designed for structure-function studies. This construct offers several advantages that remain difficult or impossible with membrane-embedded GPCRs: 1) In solution, wsMOR can be characterized at high concentrations and can therefore be applied to multiple techniques, including neutron scattering and high-resolution solution NMR. 2) Its aqueous solubility also supports precise biophysical ligand profiling (ITC, SPR, MST) at concentrations exceeding 100 μM. 3) In addition, the absence of high detergent amounts facilitates fragment and covalent ligand soaking or crystallization without detergent interference, and exposes the entire intracellular surface for site-specific conjugation and HDX-MS.

Our results show that wsMOR is folded and thermally stable outside its native membrane environment. Although the unfolding experiments indicate proper folding, the thermal transitions appear to exhibit limited cooperativity, which could be explained by the lack of a native lipid bilayer and the presence of the anionic detergent SDS. These conditions may leave a subset of receptors in a molten globule-like state, retaining substantial α-helical secondary structure but lacking the fully native tertiary fold (45). Nevertheless, the receptor selectively binds opioid ligands, thereby inducing conformational changes. In solution, wsMOR predominantly exists as a monomer, but at higher concentrations, oligomer-like assembly states are observed. At 0.01% SDS (0.05 CMC), where micelles would not form, the detergent preferentially associates with exposed hydrophobic and cationic regions of the receptor, forming protein-detergent complexes in solution. This behavior is supported by previous studies showing that the anionic groups of SDS first interact with cationic amino acid residues, followed by association with exposed hydrophobic regions on proteins (32, 46), leading to increases in apparent particle size even below the CMC. Consistent with this interpretation, the MD simulation data, combined with a GA, revealed configurations in which wsMOR monomers are separated by ∼ 30 Å (Figs. S5 and S7), distances incompatible with stable protein-protein interfaces and indicative of detergent-mediated organization. In agreement with the AUC results, the SANS model predicts that the monomer is the most populated, followed by the tetramer-like species (10.5%), the second-most populous oligomer-like state, compared to the trimer (3.7%). Regarding the agreement with the experimental *P(r)* in the polydisperse regime between 50 to 80 Å, the GA-SANS method selects representative structures but does not fully capture the full polydispersity of the solution, which is a potential limitation of the approach.

Our smFRET experiments revealed that wsMOR can undergo conformational changes upon ligand binding and in the presence of G protein. In the absence of agonists or G proteins, wsMOR spontaneously samples multiple inactive and active conformations in its unliganded state, indicating basal activity, consistent with previous findings for β_2_AR (47). In this unliganded condition, a significant population (32.7%) of receptors adopts an intermediate-inactive conformation, where the intracellular half of TM6 is not fully open (Fig. 5*C*). This observation suggests that MOR can engage in ligand-independent interactions and pre-couple with G proteins, a notion supported by previous MD simulation data (19) and consistent with the basal signaling activity of MOR in the absence of ligands (48). Stimulation with the full agonist DAMGO resulted in a marked increase in the population of active states and a concurrent decrease in inactive states (Fig. 5*D*), indicating that DAMGO promotes an active receptor conformation. Importantly, the DAMGO-activated receptor primarily stabilizes an intermediate-active conformation rather than the fully activated state, in agreement with previous studies on other class A GPCRs, including MOR (17, 21, 44).

The PAM BMS-986122, when combined with DAMGO, further increased the relative population of the low-FRET active state, surpassing DAMGO alone, and was followed by a concurrent destabilization of the intermediate-active and intermediate-inactive states (Fig. 5*E*). This finding is in agreement with a recent NMR study, which showed that BMS-986122 destabilizes intermediate-active states and promotes a fully activated conformation by rearranging the hydrophobic cluster between TM3 and TM6, locking the intracellular half of TM6 in an outwardly displaced position (21). In addition to the effects of agonists and PAMs, our results also revealed that wsMOR forms a complex with Gα_i1_ (Fig. 5*F*). Gα_i1_ binding was more effective at stabilizing the low-FRET conformation and concurrently destabilizing the intermediate-inactive conformation when compared to DAMGO alone and the DAMGO and BMS-986122 combination. The increase in the low-FRET (0.33) population, accompanied by a decrease in the intermediate-FRET (inactive) state, indicates adoption of active-like conformations in the presence of G protein. However, the unexpected increase in the high-FRET (0.78) population upon G protein binding (Fig. 5*F*) likely corresponds to a subset of non-functional receptors in a molten globule-like state that are unable to bind ligand and couple to G protein.

The low-FRET conformation associated with PAM and Gα_i1_ binding corresponds to an additional ∼7 Å outward displacement of the intracellular half of TM6 relative to TM4, a degree of opening that has not been observed in available structures or smFRET studies of MOR. This extended conformation may reflect the removal of constraints present in membrane-embedded or micelle-reconstituted systems, including the absence of lateral membrane pressure, which could permit greater structural relaxation (49), and the use of minimal SDS (∼0.01%) for stabilization, avoiding micelle formation that could otherwise restrict TM6 mobility. The lack of specific lipid–protein interactions present in a native bilayer (*e.g.*, anionic lipids or cholesterol) may further alter stabilizing contacts and shift the receptor’s conformational equilibria (50, 51). Rather than representing a non-native artifact, this state reflects a degree of TM6 opening that becomes accessible in the absence of these constraints. Although measured inter-fluorophore distances may also include contributions from local flexibility at the intracellular helix ends due to flexible fluorophore linkers (52), the selective stabilization of this state by known activators (PAM and Gα_i1_) is consistent with its classification as an active-like conformation. Accordingly, our conclusions are limited to demonstrating that wsMOR can access a more extended TM6 displacement when bilayer or micellar confinement is removed.

Biophysical and computational studies of class A GPCRs, such as β_2_AR, cannabinoid receptor 1 (CB1R), and adenosine A_2A_ receptor (A_2A_AR), have shown that agonist binding shifts the receptor towards active conformations by destabilizing the inactive states (44, 53). In the absence of G protein coupling, these receptors often remain conformationally dynamic, sampling a range of several conformational states. In line with these observations, the quasi-elastic neutron scattering data indicate that binding of the agonist DAMGO to wsMOR results in a local stabilizing effect. Specifically, DAMGO increases the activation energy associated with picosecond-scale internal motions, indicating suppression of localized side-chain fluctuations within the receptor. This dynamic shift reflects a more rigid internal environment upon agonist binding, even in the absence of downstream signaling partners like G proteins. These findings are also consistent with the thermal shift data, which show that wsMOR exhibits enhanced thermal stability in the presence of agonists. In addition, our smFRET analysis reveals an increased population of receptors occupying an active-like conformation upon DAMGO binding, supporting the interpretation that local flexibility is reduced in the agonist-bound state. Interestingly, although internal dynamics are significantly reduced upon DAMGO binding, the global translational diffusion of the receptor does not change between the unliganded and agonist-bound states. This observation indicates that receptor activation involves internal stabilization without significantly affecting its overall hydrodynamic properties or shape.

### Scope and limitations of wsMOR

Although the native membrane environment and cholesterol are critical for MOR biogenesis, stability, and its full signaling repertoire, the water-soluble variant shows that the core seven-transmembrane fold, orthosteric ligand-binding sites, native-like dynamics, and even G protein interactions can be maintained independently of the lipid bilayer once folding is complete. However, the absence of native lipids means that membrane-dependent aspects of ligand binding, allostery, conformational dynamics, and receptor–protein interactions cannot be fully captured with this construct. In this context, our findings complement rather than contradict the established membrane dependence of GPCR function; wsMOR preserves several intrinsic structural and dynamics properties of MOR, but lipid-specific contributions to signaling and allosteric regulation will still require membrane-based systems. In that regard, wsMOR is well-suited for evaluating structural features, intrinsic and global dynamics, ligand effects, and G protein coupling outside a membrane environment, while questions involving lipid-mediated modulation, cholesterol-dependent stabilization, and complete activation mechanisms warrant further investigation in lipid-based environments. At the same time, while wsMOR can bind Gα_i1_ and adopt active-like conformations, functional signaling was not directly measured in this study. Consequently, the extent to which wsMOR can activate native signaling pathways, reproduce pharmacology, or exhibit the same bias profiles as the native receptor remains unknown.

In summary, this study characterizes the conformational dynamics of wsMOR under ligand- and G protein-bound conditions, complementing previous observations in nanodisc- or micelle-stabilized wild-type MOR (20, 54). Ligand binding reduces internal flexibility, consistent with stabilization of local motions even in the absence of a membrane or G protein. G protein engagement preferentially stabilizes a low-FRET conformation with an outward TM6 displacement greater than that seen in resolved structures, representing an extension of the active-like ensemble that has not been previously observed. Positive allosteric modulators shift the receptor population toward this extended, low-FRET state, to an extent intermediate between agonist alone and G protein binding, highlighting modulation of the conformational landscape. These findings provide mechanistic insight into GPCR dynamics, demonstrate the utility of engineered soluble GPCR variants for probing conformational mechanisms and ligand effects in aqueous environments, and contribute to our understanding of how pharmacological agents like ligands and G proteins differentially stabilize receptor conformations.

## Experimental procedures

### wsMOR expression and purification

The gene encoding the water-soluble variant of the N-terminally truncated human MOR (66–400) was expressed in *E. coli* BL21 (DE3) competent cells (Thermo Fisher Scientific; EC0114) and purified as described previously, with some modifications (55, 56). A hexahistidine tag and a FLAG tag were added to the amino terminus to facilitate purification and further characterization. Cell pellets were resuspended in lysis buffer (50 mM Tris-HCl, 1 M urea, pH 8.0). To this, EDTA was added to a final concentration of 1 mM, followed by 6 mM 2-mercaptoethanol (2-ME), 1% Triton X-100, and hen egg lysozyme at a concentration of 1 μg per OD of cells. The suspension was mixed thoroughly and incubated at room temperature (RT) for 20 min. Subsequently, MgCl_2_ was added to a final concentration of 3 mM, along with 100 units of benzonase. The suspension was swirled gently and incubated for 5 min at RT before centrifugation at 10,000 rpm for 20 min at 20 °C using an SS-34 rotor. The resulting pellet was washed twice with PT buffer (100 mM phosphate, 10 mM Tris, pH 8.0) containing 40 mM 2-ME. The pellet was dispersed using a pipette and homogenized with sonication before filling the tube with PT buffer. The suspension was mixed by inversion and centrifuged under the same conditions, and the supernatant was discarded. A third wash was performed using PT buffer supplemented with 1% sodium deoxycholate, 1% Nonidet P-40, and 10 mM 2-ME. The pellet was dispersed as above, topped up with PT buffer, and centrifuged at 10,000 rpm for 20 min at 20 °C. The final pellet was resuspended in PT buffer containing 0.2% SDS, 5% glycerol, and 40 mM 2-ME. The two suspensions were mixed thoroughly by repeatedly pouring back and forth between tubes and then rocking gently overnight at 4 °C until the solution appeared nearly clear. The clarified suspension was transferred into two 38 ml Oak Ridge Centrifuge Tubes (Thermo Fisher Scientific) and centrifuged at 12,000 rpm for 20 min at 20 °C using an SS-34 rotor. The solubilized receptor supernatants were recovered and purified using an ÄKTA start FPLC system equipped with a HisTrap HP column (Cytiva) at RT. To isolate wsMOR, the supernatants were loaded onto a pre-packed Ni^2+^-NTA affinity column pre-equilibrated with binding buffer (PT buffer, pH 8.0, 5% glycerol, 5 mM 2-ME, 0.01% SDS). The column was then washed with 10 column volumes of wash buffer (PT buffer, pH 8.0, 5% glycerol, 5 mM 2-ME, 0.01% SDS, and 10 mM imidazole) to remove nonspecifically bound proteins. The receptor was eluted with elution buffer (PT buffer, pH 8.0, 5% glycerol, 5 mM 2-ME, 0.01% SDS, and 500 mM imidazole). The eluted protein was collected in several fractions, and its purity was assessed using SDS-PAGE. Fractions containing wsMOR were pooled and dialyzed overnight at 4 °C against dialysis buffer (PT buffer, pH 8.0, 5% glycerol, 5 mM 2-ME, 0.01% SDS) to remove excess imidazole. The purified wsMOR was stored at 4 °C for downstream applications.

### Expression and purification of human Gα_i1_

The recombinant full-length human Gα_i1_ protein was purified by standard protocols with minor modifications, as described previously (57). The plasmid encoding recombinant human Gα_i1_ with an N-terminal hexahistidine tag and a SUMO tag was transformed into *E. coli* BL21 (DE3) (Thermo Fisher Scientific; EC0114) competent cells. The cells were cultured in Luria-Bertani Broth medium supplemented with kanamycin and incubated at 37 °C overnight. Once the culture reached an OD600 of 0.6 to 0.8, protein expression was induced by adding 1 mM IPTG, followed by a 4-h incubation at 37 °C. The culture was harvested by centrifugation at 10,000 rpm for 10 min at 4 °C and stored at −80 °C until further use. Cell pellets were thawed in lysis buffer (25 mM sodium phosphate buffer, pH 7.4, 50 μM MgCl2, 500 μM (tris(2-carboxyethyl)phosphine) (TCEP), 50 μM GDP, and EDTA-free protease inhibitor tablets [Thermo Fisher Scientific; A32955]) and lysed by sonication. The lysate was then clarified by centrifugation at 17,500 rpm for 30 min at 4 °C. The supernatant was incubated with 1 ml (2 ml 50/50 slurry) of HisPur Ni2+-NTA Resin (Thermo Fisher Scientific; 88,221) pre-equilibrated with wash buffer A (25 mM sodium phosphate buffer, pH 7.4, 50 μM MgCl_2_, 500 μM TCEP, 50 μM GDP, 10 mM imidazole) for 10 min at 4 °C with gentle rotation. The resin was washed five times with wash buffer A, followed by five washes with wash buffer B (25 mM sodium phosphate buffer, pH 7.4, 50 μM MgCl2, 500 μM TCEP, 50 μM GDP, 30 mM imidazole). The protein was eluted with elution buffer pH 7.4 (wash buffer with 250 mM imidazole). The SUMO tag was cleaved by the addition of 0.15 mg *Chaetomium thermophilum* SUMO protease, and the eluate was dialyzed overnight at 4 °C in a wash buffer without imidazole. The cleaved protein was subsequently purified by reverse-nickel chromatography.

### CD

CD spectra were measured with 0.3 mg/ml of the purified receptor in a buffer containing PT buffer, pH 8.0, 130 mM NaCl, and 0.01% SDS. A volume of 100 μl of the purified receptor was used for each experiment. The measurements were performed in a Jasco J-185 CD Spectropolarimeter over 198 to 260 nm wavelength with a scan speed of 1 nm/s, step size of 1 nm, an averaging time of 4 s, and a 1 mm path length. The obtained CD spectrum was analyzed and fitted using BestSel (58). Igor Pro 8 (WaveMetrics, Lake Oswego, OR, USA) was used for data analysis and visualization.

### Fluorescence thermal shift assay

Thermal shift assays were performed as described previously (27) with a few modifications. A stock solution of N-[4-(7-diethylamino-4-methyl-3-coumarinyl) phenyl]maleimide (CPM; Invitrogen) was prepared by dissolving it at a concentration of 4 mg/ml in DMSO (Sigma). The CPM dye stock solution was diluted 1:40 in CPM buffer (20 mM Hepes, pH 8, 200 mM NaCl, and 0.01% SDS) and incubated for 5 min at RT. A volume of 10 μl of the diluted CPM dye was added and thoroughly mixed with 4 μM of the receptor in a final volume of 130 μl. The reaction mixture was then incubated for 30 min in the dark at 4 °C. For ligand-binding experiments, 40 μM of DAMGO or glucagon was added to the receptor and incubated for 1 h in the dark at 4 °C. The assays were conducted in Cary Eclipse Fluorescence Spectrophotometer (Agilent Technologies) using quartz cuvettes (Starna Cells, Inc.), with a ramp rate of 2 °C/min over a temperature range from 20 °C to 90 °C. Fluorescence intensity measurements were taken at an excitation wavelength of 387 nm and an emission wavelength of 463 nm. The data generated were analyzed using GraphPad Prism 10 (https://www.graphpad.com), and the raw data were fitted to a Boltzmann sigmoidal curve to ascertain the melting temperature (Tm).

### Analytical ultracentrifugation

Sedimentation velocity experiments were performed at 20 °C and 50,000 rpm using an Optima Analytical Ultracentrifuge (Beckman Coulter equipped with an An-50 Ti rotor. Protein concentrations of 0.5, 2.5, and 10 mg/ml were analyzed. Briefly, 390 μl of each wsMOR sample was loaded into analytical cells with standard 12-mm double-sector centerpieces equipped with sapphire windows, and 400 μl of matched analysis buffer (PT buffer, pH 8.0, 5% glycerol, 5 mM β-mercaptoethanol, 0.01% SDS) was loaded into the reference sector. Data were acquired using absorbance detection at 280 nm and interference (IF) optics. Approximately 500 scans were collected per sample with a radial increment of 10 μm, and IF data were acquired at 2-min intervals. Data were analyzed using the continuous c(s) distribution model in SEDFIT (59), fitting for the frictional ratio (1.29225), meniscus position (6.0533 cm), time-invariant noise, and radial-invariant noise, with a confidence level of 0.68. The sedimentation coefficient (s) range of 0 to 15 S was evaluated at a resolution of 300. The buffer density and viscosity were set to 1.02642 g/ml and 0.01107 P, respectively, and the partial specific volume (v) of wsMOR was set to 0.7311 ml/mg. Final distributions were visualized and graphically presented using GUSSI (60).

### Small-angle scattering data collection and analysis

SAXS data were collected using a Rigaku BioSAXS-2000 instrument. Purified unliganded wsMOR was dialyzed overnight at 4 °C against dialysis buffer (PT buffer, pH 8.0, 5% glycerol, 5 mM 2-ME, 0.01% SDS). Protein samples were prepared at concentrations of 0.7 mg/ml and 2.76 mg/ml in dialysis buffer and centrifuged at 13,000 rpm for 30 min to remove aggregates. SAXS measurements were performed by loading the soluble fraction (supernatant) into the sample exposure cell set at 4 °C. Scattering data were collected for 10 min per frame for 2 h. Buffer scattering profiles were collected before and after each sample measurement and the final protein scattering curves were scaled by concentration. Data reduction was performed using Rigaku BioSAXS-2000 software (https://rigaku.com). Background subtraction, Guinier, and pair-distance distribution function, *P(r)* analyses were carried out using BioXTAS RAW (61). The *P(r)* was determined using the indirect Fourier transform method in GNOM (62), which provides real-space structural information about unliganded wsMOR.

Small-angle neutron scattering (SANS) experiments were performed at the Bio-SANS beamline (CG-3) of the High Flux Isotope Reactor at Oak Ridge National Laboratory (ORNL) (63). Data were collected using a three-detector array. The small-angle detector was positioned at 7.0 m from the sample, the mid-range detector at 4.0 m, and −2.7° rotation from the direct beam, and the high-angle detector at 1.13 m and +7.25° rotation from the direct beam. This configuration covered a momentum transfer range of 0.007 < Q < 0.85 Å^−1^ using neutrons with a wavelength of 6.44 Å and a relative wavelength spread (Δλ/λ) of 0.123. The momentum transfer (*Q*) is defined as *Q* = *4π·sin(θ)/λ*, where 2θ is the scattering angle and *λ* is the neutron wavelength. The Panel Scan feature (64) of the data acquisition system was used to control the instrument during measurement. Data reduction included corrections for instrument background, detector sensitivity, and instrument geometry, followed by circular averaging to 1D scattering profiles using the facility drt-SANS (65) software. Reference measurements, including empty beam, beam center, and buffer backgrounds, were measured and applied during data reduction processing using drt-SANS. Samples were measured at RT using a 1 mm quartz cylindrical cuvette (Hellma).

### Structural prediction and computational modeling

The structure of the N-terminally truncated wsMOR was predicted using the AlphaFold3 server (https://alphafoldserver.com/) (66) and the predicted model with the highest rank was selected for further analysis. Visualization and structural analyses were carried out using UCSF ChimeraX (67). To enhance clarity and simplify the representation, the disordered C-terminal tail was excluded from the figures. The wsMOR was superimposed onto the antagonist-bound mouse MOR (PDB ID: 4DKL) and the BilboMD/GA model, and RMSDs were calculated using UCSF ChimeraX. Surface hydrophobicity patches were also rendered on the protein models for further analysis.

AlphaFold3 structures were generated for the monomer, dimer, trimer, and tetramer forms of wsMOR in the absence of detergent. These structures were entered into the BilboMD-SANS (68) tool at the SIBYLS beamline (https://bilbomd.bl1231.als.lbl.gov/). The constraints for the monomer were generated using AlphaFold3’s PAE values in the BilboMD “PAE Jiffy” tool. For the dimer, trimer, and tetramer simulations, only one of the monomers was fixed in space, and the same residue restraints were applied as above. The SANS intensities were calculated from the BilboMD sampled conformers for each oligomer simulation at 98% D_2_O with 501 q-points from 0 to 0.5 Å^−1^ using Pepsi-SANS (69). After completing the BilboMD-SANS runs, the conformers were collected from the SIBYLS server and combined into one large ensemble with 1400 monomers, 1200 dimers, 1000 trimers, and 1000 tetramers. The combined ensemble was then refined using a genetic algorithm (GA-SAS) (70). The GA selects a set of *N* structures from the combined ensemble that optimizes the reduced χ^2^ agreement to experiment as a linear combination of the individual scattering curves. Up to N = 5 structures per ensemble were tested, with randomized starting parents over five iterations. Each iteration consisted of 100 generations and was run on the ORNL Compute and Data Environment for Science cluster. The GA returned the best set of structures as well as the best model SANS profile for each ensemble size and iteration. The best result was a four-state ensemble with a χ^2^ of 1.92.

For the long-timescale all-atom MD simulations, the wsMOR structure was prepared using the CHARMM36 all-atom force field (71) and solvated in TIP3P water with 0.15 M sodium and chloride ions, balanced such that the system was at overall neutral charge. CHARMM-GUI (72, 73) was used for system setup, minimization and equilibration protocols. After minimization and equilibration, production simulation was conducted in the isothermic-isobaric ensemble using periodic boundary conditions, Langevin thermostat, and Langevin barostat, with NAMD 2.14 software (https://www.ks.uiuc.edu) (74). The simulation time was 1 μs for each wsMOR simulation. A simulation of mouse MOR (PDB: 4DKL) in a 70:30 mol % (1-palmitoyl-2-oleoyl-sn-glycero-3-phosphocholine): cholesterol lipid bilayer (as cholesterol is an important interaction partner of opioid receptors), TIP3P water, and 0.15 M sodium and chloride ions for electroneutrality was also conducted with a simulation time of 649 ns. Visual Molecular Dynamics 2.0a5 (75) was used for analysis and figure generation. Root-mean-square deviation of alpha carbons was calculated every 20 ps.

### Quasi-elastic neutron scattering (QENS)

QENS measurements were conducted using the Backscattering Silicon Spectrometer (BASIS) (34) at the Spallation Neutron Source at ORNL. Purified wsMOR was dialyzed overnight at 4 °C against dialysis buffer (PT buffer, pH 8.0, 5% glycerol, 5 mM 2-ME, 0.01% SDS) and concentrated to ∼55 mg/ml using a 15 ml 30 kDa MWCO centrifugal filter (MilliporeSigma; UFC901024). For QENS measurements, wsMOR at ∼55 mg/ml was dialyzed against a deuterated dialysis buffer (100 mM sodium phosphate pD 7.6, 10 mM Tris-D_11_, 5% D-glycerol, 0.01% SDS-D_25_, 5 mM 2-ME) prepared in 100% D_2_O. This process was repeated four additional times with fresh deuterated dialysis buffer to ensure that ∼99.98% of exchangeable hydrogens in the sample were replaced with deuterium. The deuterated sample was loaded into a mini dialysis membrane and equilibrated with the deuterated dialysis buffer under gentle rotation at 4 °C. The resulting buffer was used as the matched deuterated buffer for QENS measurements. The volume fraction of wsMOR in the sample was ∼4.09%. For DAMGO-bound wsMOR experiments, a five-fold molar excess of deuterated DAMGO was incubated with ∼46.7 mg/ml wsMOR for 1 h at RT before QENS analysis. Samples were loaded into standard annular aluminum holders equipped with inserts to achieve a sample thickness of approximately 0.25 mm. The sample temperature was controlled using a closed-cycle refrigerator. Measurements were performed at 310, 300, 290, and 280 K, in a standard spectrometer configuration (33, 34), providing an instrument resolution of approximately 3.4 μeV (full width at half-maximum) and an accessible energy transfer range of ±100 μeV. The resolution spectra were measured at the baseline temperature of 15 K. The buffer signal was measured separately at each temperature and subtracted from the raw QENS spectra before data analysis. QENS data were reduced and analyzed using the Mantid (76) and DAVE (77) software, respectively.

### Fluorophore labeling of wsMOR for smFRET

wsMOR with cysteine mutations on TM4 (R184C) and TM6 (R278C) was diluted in labeling buffer containing PT buffer, pH 8.0, 130 mM NaCl, and 0.01% SDS, then incubated with a ten-fold molar excess of Lumidyne 555-maleimide (LD555-MAL; donor) and a twenty-fold molar excess of Lumidyne 655-maleimide (LD655-MAL; acceptor) [Lumidyne Technologies]. The solution was gently rotated in the dark at 4 °C for 3 h. To remove excess dye and concentrate the protein, the solution was loaded onto an Amicon Ultra-0.5 ml 10 kDa MWCO centrifugal filter (MilliporeSigma #UFC501096) pre-equilibrated with labeling buffer and centrifuged at 10,000 rpm for 10 min. The labeling buffer was added to the filter column and centrifuged three times at 10,000 rpm for 10 min each time. The labeling efficiency was determined using a NanoDrop One^C^ spectrophotometer by measuring absorbance at 280 nm for the total protein, 555 nm for the LD555-MAL dye, and 655 nm for the LD655-MAL dye.

### Spectroscopy and Förster radius determination of labeled wsMOR

Steady-state absorption and fluorescence measurements were performed on purified wsMOR labeled with a five-fold molar excess of either LD555 or LD655 (Fig. S8). Measurements were conducted at RT using a Cary Eclipse Fluorescence Spectrophotometer (Agilent Technologies). Labeled samples were prepared at a final concentration of 1 μM in labeling buffer (PT buffer, pH 8.0, 130 mM NaCl, and 0.01% SDS). For the fluorescence emission spectrum, LD555-labeled samples were excited at 517 nm. Emission was recorded from 520 to 750 nm in 1 nm increments with a 0.1 s averaging time. To record excitation spectra of LD655-labeled samples, the emission monochromator was set to zero-order mode (*i.e.*, allowing all emitted wavelengths to pass), and excitation wavelengths were scanned from 400 to 750 nm. All measurements were acquired in a 10 mm pathlength quartz cuvette (Starna Cells, Inc.).

The Förster radius ($R_{0}$, in Å) for labeled wsMOR was calculated using the equation:

(1) $$R_{0}=0.211\cdot \sqrt[{6}]{\kappa^{2}\;\eta^{-4}\;Q_{D}\;J\left(\lambda \right)}$$

where $\kappa^{2}$ is the dipole orientation factor, η is the refractive index of the medium, $Q_{D}$ is the quantum yield of the donor, and J(λ) is the spectral overlap integral (in M^−1^ cm^−1^ nm^4^) between the donor emission and absorption spectra, defined as:

(2) $$J\left(\lambda \right)=\int_{0}^{\infty }F_{D}\left(\lambda \right)\varepsilon_{A}\left(\lambda \right)\lambda^{4}d\lambda /\int_{0}^{\infty }F_{D}\left(\lambda \right)d\lambda$$

where $F_{D}\left(\lambda \right)$ is the normalized emission spectrum of the donor, $\epsilon_{A}\left(\lambda \right)$ is the molar extinction coefficient of the acceptor, and λ is the wavelength (78, 79).

### Total internal reflection fluorescence-based single-molecule FRET

Single-molecule fluorescence studies were conducted at RT using a custom-built prism-based TIRF imaging system based on an Olympus IX73 inverted microscope equipped with a 60× water-immersion objective (Olympus, 1.49 numerical aperture) as described previously (4). Quartz slides (G. Finkenbeiner) and coverslips were passivated with polyethylene glycol (m-PEG-SVA) and 3% (w/w) biotin-PEG-SVA (Laysan Bio Inc., Arab, AL) to minimize non-specific binding. For unliganded wsMOR measurements, the N-terminal FLAG-tagged receptor was immobilized on a quartz slide *via* a biotin-streptavidin-FLAG antibody interaction, forming a stable surface attachment. A sample chamber was prepared using double-sided tape, and reagents were injected into the channel in the following order: 0.02 mg/ml streptavidin, imaging buffer A (20 mM sodium phosphate, pH 8, 130 mM NaCl, 0.01% SDS, 2 mM Trolox), 33 nM biotin-conjugated anti-FLAG tag antibody, imaging buffer A, fluorophore-labeled unliganded wsMOR, imaging buffer A, and an oxygen scavenger system containing 2 mM Trolox, 5 mM protocatechuic acid, 3 U/ml protocatechuate-3,4-dioxygenase (Oriental Yeast). The sample was illuminated with a 532 nm laser (CrystaLaser), and fluorescence emission from donor and acceptor fluorophores was collected on an EMCCD camera (Andor Technology) with exposure times of 100 ms. Images were recorded using a custom single-molecule data acquisition program (80). The data collected was analyzed using scripts written in IDL software (https://www.nv5geospatialsoftware.com/Products/IDL; Harris Geospatial Solutions, Inc.). The source code used for data acquisition and extraction is available in a repository at https://github.com/Ha-SingleMoleculeLab. We used custom-built MATLAB scripts for further smFRET data analysis and Igor Pro macros to perform the histogram analysis as described previously (81). For each condition tested, smFRET trajectories were pooled and analyzed separately using STaSI to identify the corresponding conformational states (37).

DAMGO-bound wsMOR measurements were conducted in a similar manner to the unliganded condition. Specifically, the receptor was incubated with a saturating concentration of DAMGO (10 μM) prepared in imaging buffer A for 20 min at RT before imaging. For experiments involving GDP-bound Gα_i1_, surface-immobilized wsMOR was first incubated with imaging buffer B (20 mM sodium phosphate, pH 8, 130 mM NaCl, 0.01% SDS, 1 mM MgCl_2_, 50 μM GDP, 10 μM DAMGO, 2 mM Trolox) to facilitate ligand binding. Subsequently, 8 μM Gα_i1_ diluted in imaging buffer B supplemented with oxygen scavenger system was introduced into the chamber and incubated for 30 min to allow for protein-protein interactions before imaging.

### Data availability

All data reported in this manuscript will be shared by the lead contact upon request: Rajan Lamichhane (rajan@utk.edu).

## Supporting information

This article contains supporting information (82).

## Conflict of interest

The authors declare that they have no conflicts of interest with the contents of this article.
