# Supplementary material for: Activation dynamics of a water-soluble human mu-opioid receptor
Source: J Biol Chem. 2026 Mar 20;302(5):111393. doi: 10.1016/j.jbc.2026.111393 (PMC13123355; doi:10.1016/j.jbc.2026.111393)
Supplement: Supporting information [file mmc1.docx]

Supporting Information for

**Activation dynamics of a water-soluble human mu-opioid receptor**

Eugene Agyemang, ^1^ Raegan Van Wirt, ^2^ Calixte Walls, ^3^ Thomas T. Joseph, ^4^ Sriram Tiruvadi-Krishnan, ^2^ John Grothusen, ^4^ Qiu Zhang, ^5^ Alan Hicks, ^5^ Wellington Leite, ^5^ Naresh C. Osti, ^5^ Eugene Mamontov, ^5^ Hugh M. O’Neill, ^5^ Renyu Liu, ^4^* Rajan Lamichhane, ^2^*

^1^UT-ORII Genome Science and Technology Graduate Program, University of Tennessee, Knoxville, TN 37996, USA.

^2^Department of Biochemistry & Cellular and Molecular Biology, University of Tennessee, Knoxville, TN 37996, USA.

^3^Department of Chemistry, University of Tennessee, Knoxville, TN 37996, USA.

^4^Department of Anesthesiology and Critical Care, University of Pennsylvania Perelman School of Medicine, Philadelphia, PA 19104, USA.

^5^Neutron Scattering Division, Oak Ridge National Laboratory, Oak Ridge, TN, 37830, USA.

*Correspondence: [rajan@utk.edu](mailto:rajan@utk.edu); [RenYu.Liu@pennmedicine.upenn.edu](mailto:RenYu.Liu@pennmedicine.upenn.edu)


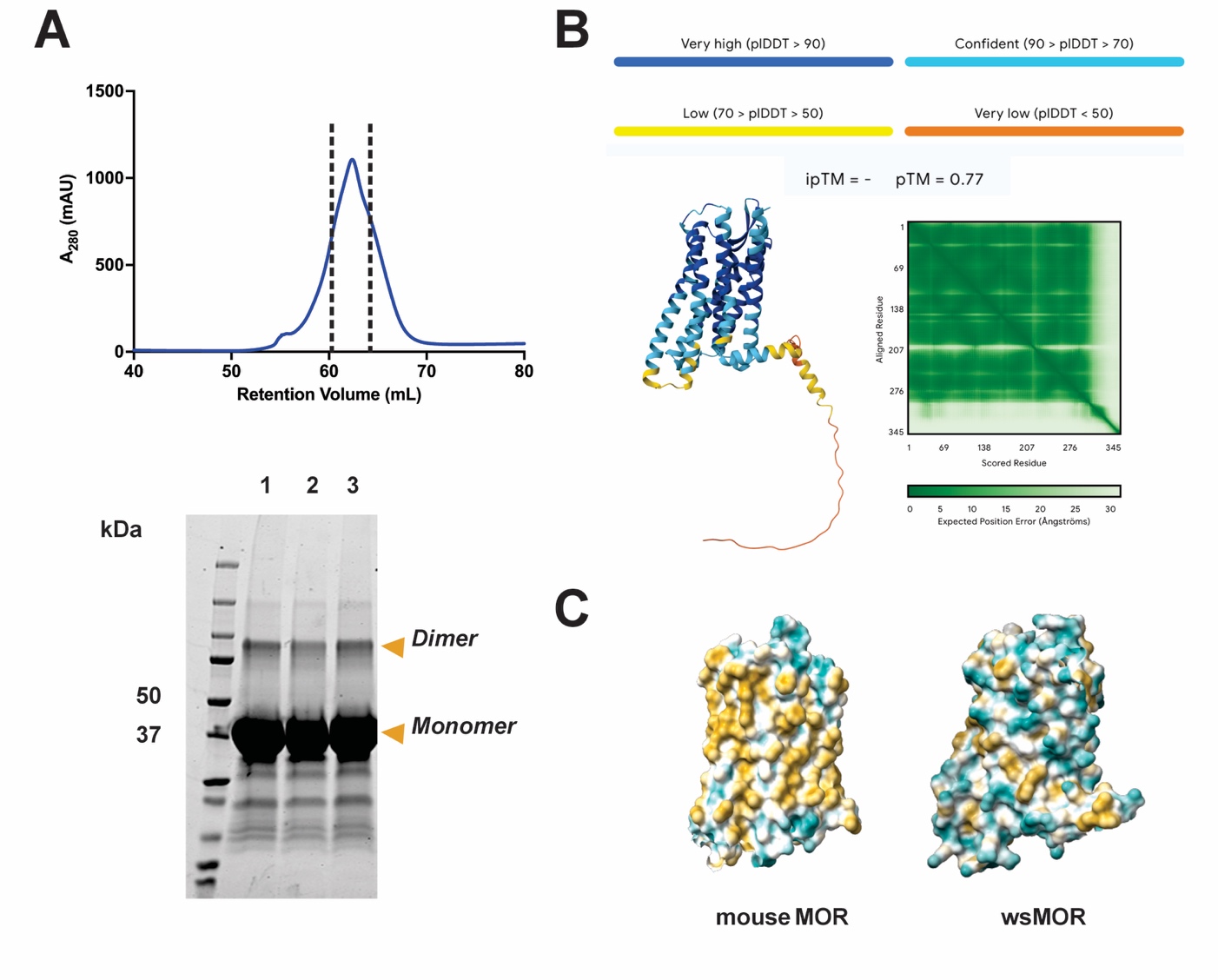


**Figure S1. Purification and characterization of the minimal cysteine wsMOR construct.** *(A)* Top, FPLC elution profile of wsMOR purified from *E. coli* BL21(DE3) cells on a HisTrap™HP 5 mL column (GE Healthcare). Bottom, Coomassie-stained, reducing SDS-PAGE gel of the elution fractions (dashed lines in top panel) from the Ni^2+^-NTA affinity purification column. The lanes labeled 1, 2, and 3 correspond to the peak elution fractions analyzed, confirming the expected molecular weight. (*B*) AlphaFold3 (AF3) prediction for wsMOR**.** The AF3 model is color-coded by predicted local distance difference test (pLDDT) confidence scores, with transmembrane regions showing high confidence (dark blue) and some unstructured regions (loops and C-terminal tail) showing lower confidence (orange to yellow). The predicted aligned error (PAE) matrix, colored from dark green (high-confidence) to white (low-confidence), represents uncertainty in inter-residue distances. Based on pLDDT and PAE, the structure is well defined for residues 1–289, while confidence is low for residues 290–345 (C terminus; white). *(C)* Hydrophobic surface analysis of mouse MOR (left) and wsMOR (right). The TM helices of MOR contain more hydrophobic (yellow) residues compared to wsMOR, which has more hydrophilic residues. The C-terminal tail of wsMOR was removed for visual clarity.

**
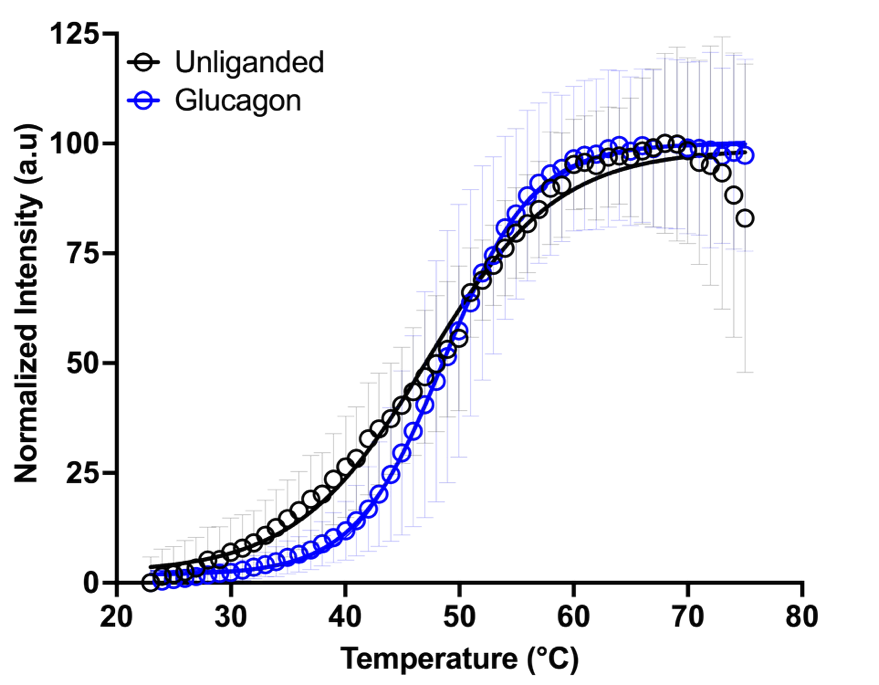
Figure S2. Interaction of wsMOR with glucagon.** Melting curves represent unliganded wsMOR (black; Tm = 47.5°C) and wsMOR in the presence of glucagon (blue; Tm = 48.6°C). The absence of a significant thermal shift upon glucagon addition indicates no detectable binding or ligand-induced stabilization of the receptor. Data are presented as the mean ± SEM for n = 2.


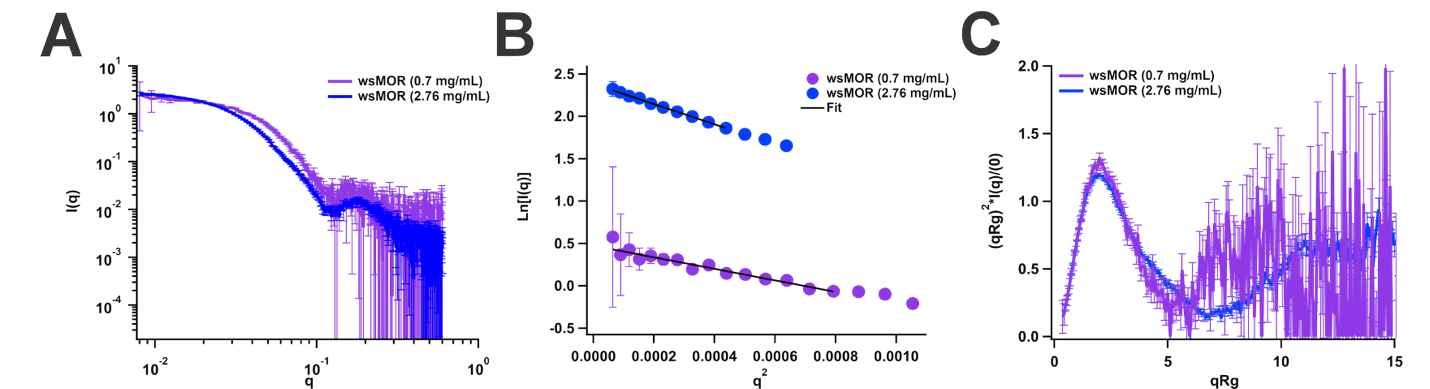


Figure S3. SAXS analysis of wsMOR. *(A)* SAXS scattering intensity profiles of unliganded wsMOR at 0.7 mg/mL (purple) and 2.76 mg/mL (blue) reveal concentration-dependent differences. Curves are scaled by concentration for direct comparison. Parameters derived from SAXS analysis are provided in Table S1. *(B)* Guinier plots corresponding to the low-q region (q*Rg < 1.3) yield *Rg* values of 43.2 Å and 59.8 Å for the 0.7 and 2.76 mg/mL samples, respectively, suggesting concentration-dependent oligomerization. *(C)* Dimensionless Kratky plots for the indicated concentrations show a bell-shaped curve, consistent with a folded, globular protein. SAXS data uncertainties are shown as solid vertical lines and were derived from counting statistics errors proportional to (N^1/2^/N), where N is the number of detector photon counts. Errors for Guinier and Kratky analyses were propagated from the SAXS data.


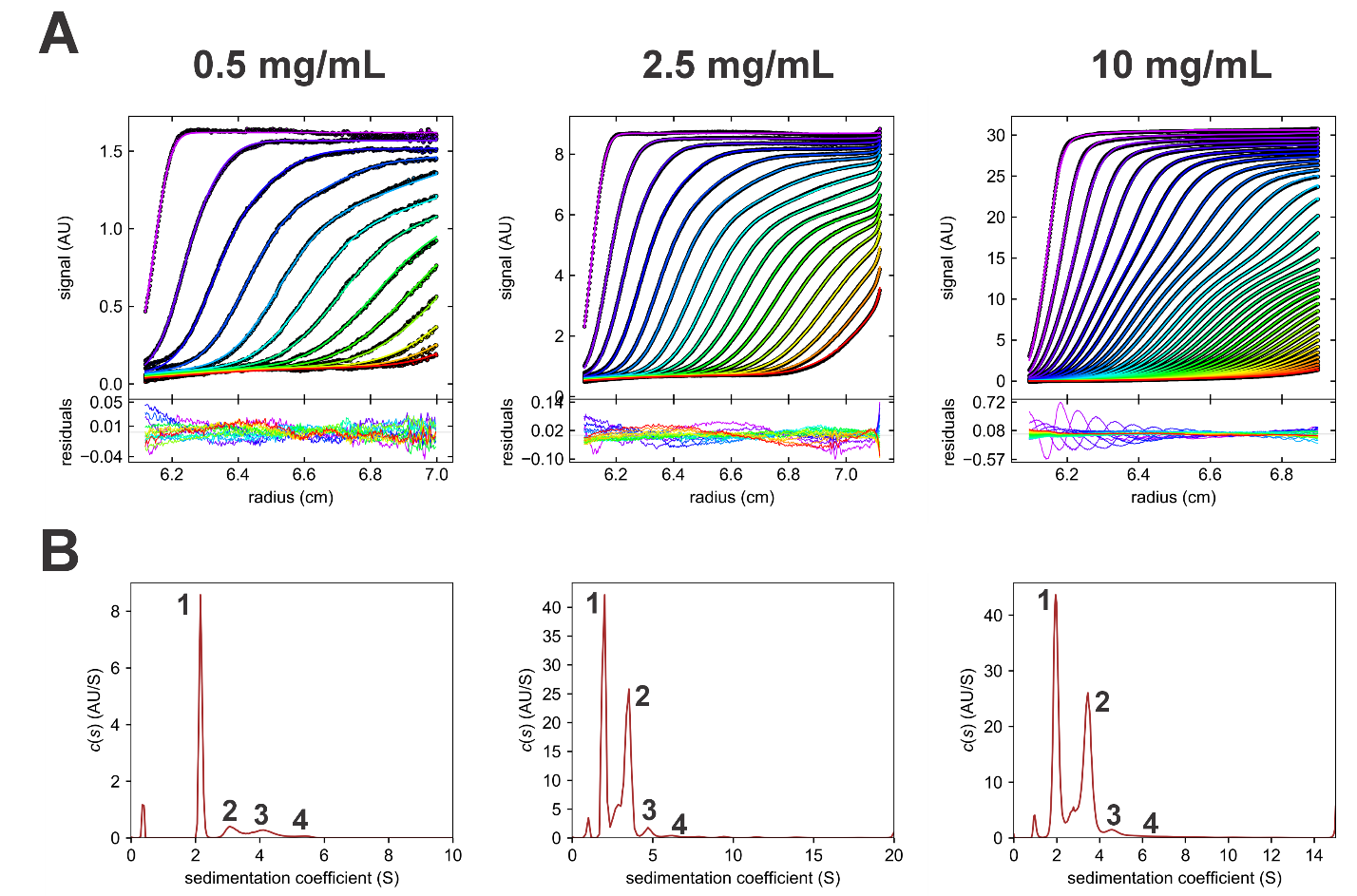


**Figure S4. Sedimentation velocity analytical ultracentrifugation (SV-AUC) analysis of purified wsMOR.** *(A)* Interference scans of purified unliganded wsMOR at concentrations of 0.5, 2.5, and 10 mg/mL were recorded at 2-minute intervals at 280 nm. Data were fitted to a continuous c(s) distribution model using the Lamm equation in SEDFIT, yielding residuals indicative of good fits. Continuous lines represent best fits to the experimental data. The initial four fringes were excluded from the analysis to minimize artifacts from large aggregates and improve model accuracy. *(B)* The calculated c(s) distribution as a function of the sedimentation coefficient (S) indicates the presence of monomeric wsMOR (peak 1) as the major species in solution across all concentrations tested. Smaller peaks (2–4) correspond to dimeric, trimeric, and tetrameric species. Sedimentation coefficients were corrected for temperature, buffer viscosity, and density. A summary of the AUC analysis is provided in Table S2**.**


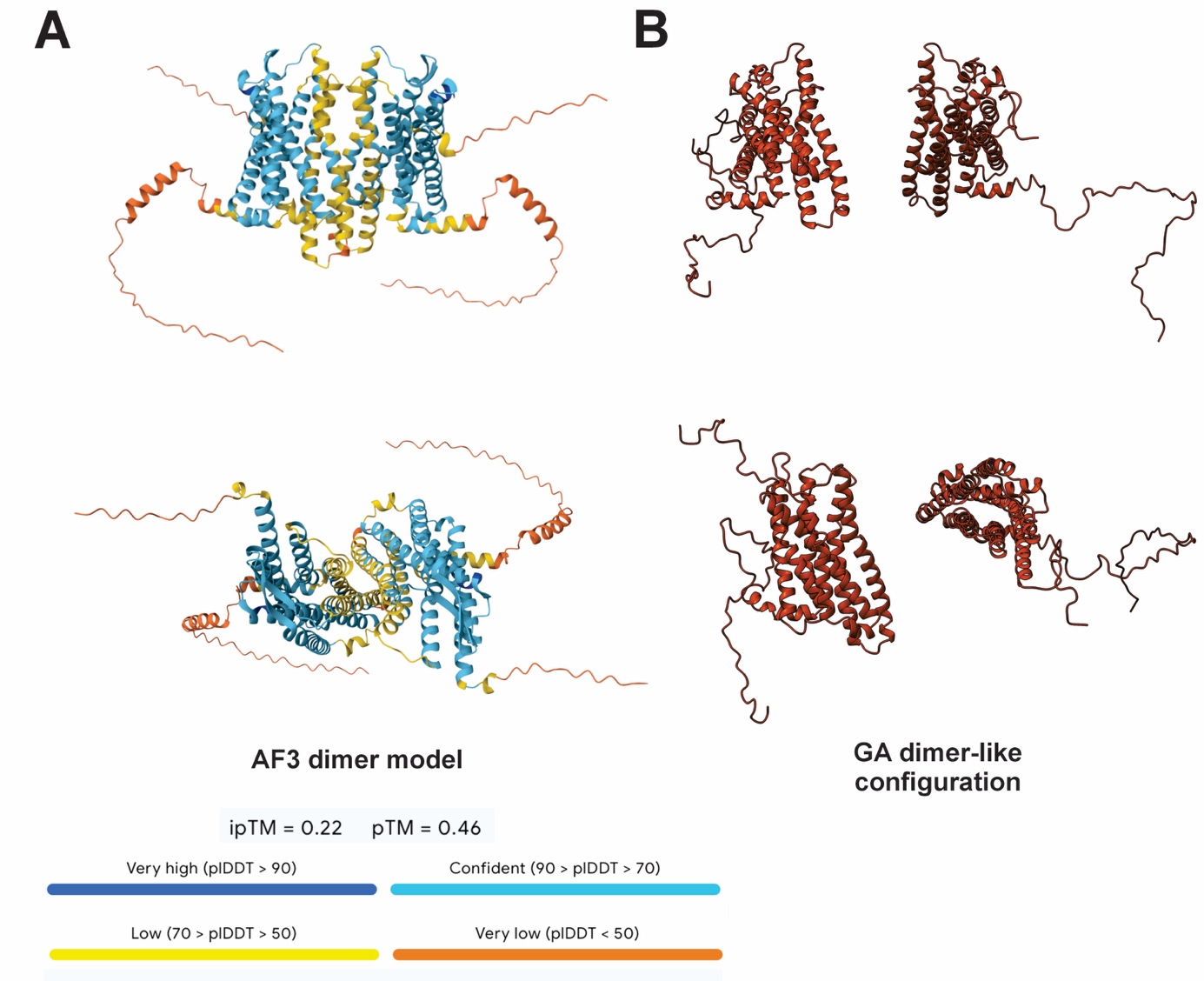


**Figure S5. AlphaFold3-predicted and GA-derived wsMOR dimer model used for SANS fitting.** (*A*) AF3-predicted wsMOR dimer, colored by per-residue confidence (pLDDT; scale shown at bottom). (*B*) GA-derived dimer-like configuration showing wsMOR subunits separated by ~30 Å. AF3-predicted models served as initial structural templates for GA-based simulations. Disordered regions correspond to the N-terminal His and FLAG tags and the C-terminal tails.


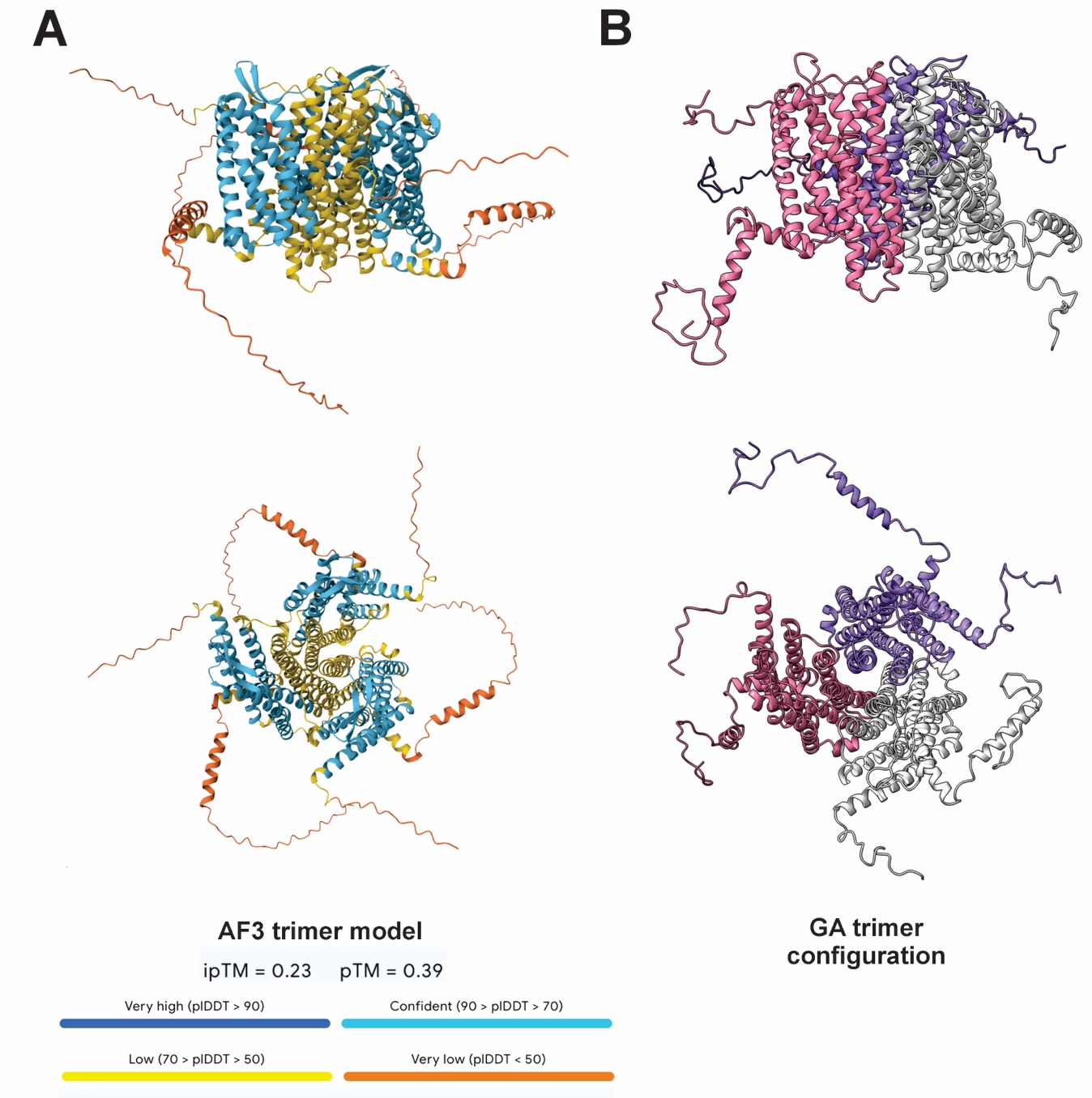


**Figure S6. AlphaFold3-predicted and GA-derived wsMOR trimer model used for SANS fitting.** *(A*) AF3-predicted wsMOR trimer, colored by per-residue confidence (pLDDT; scale shown at bottom). (*B*) GA-derived trimer model showing a compact wsMOR subunit arrangement similar to the AF3-predicted structure. AF3-predicted models served as initial structural templates for GA-based simulations. Disordered regions correspond to the N-terminal His and FLAG tags and the C-terminal tails. Individual protomers within the homotrimer are colored distinctly for visual clarity.


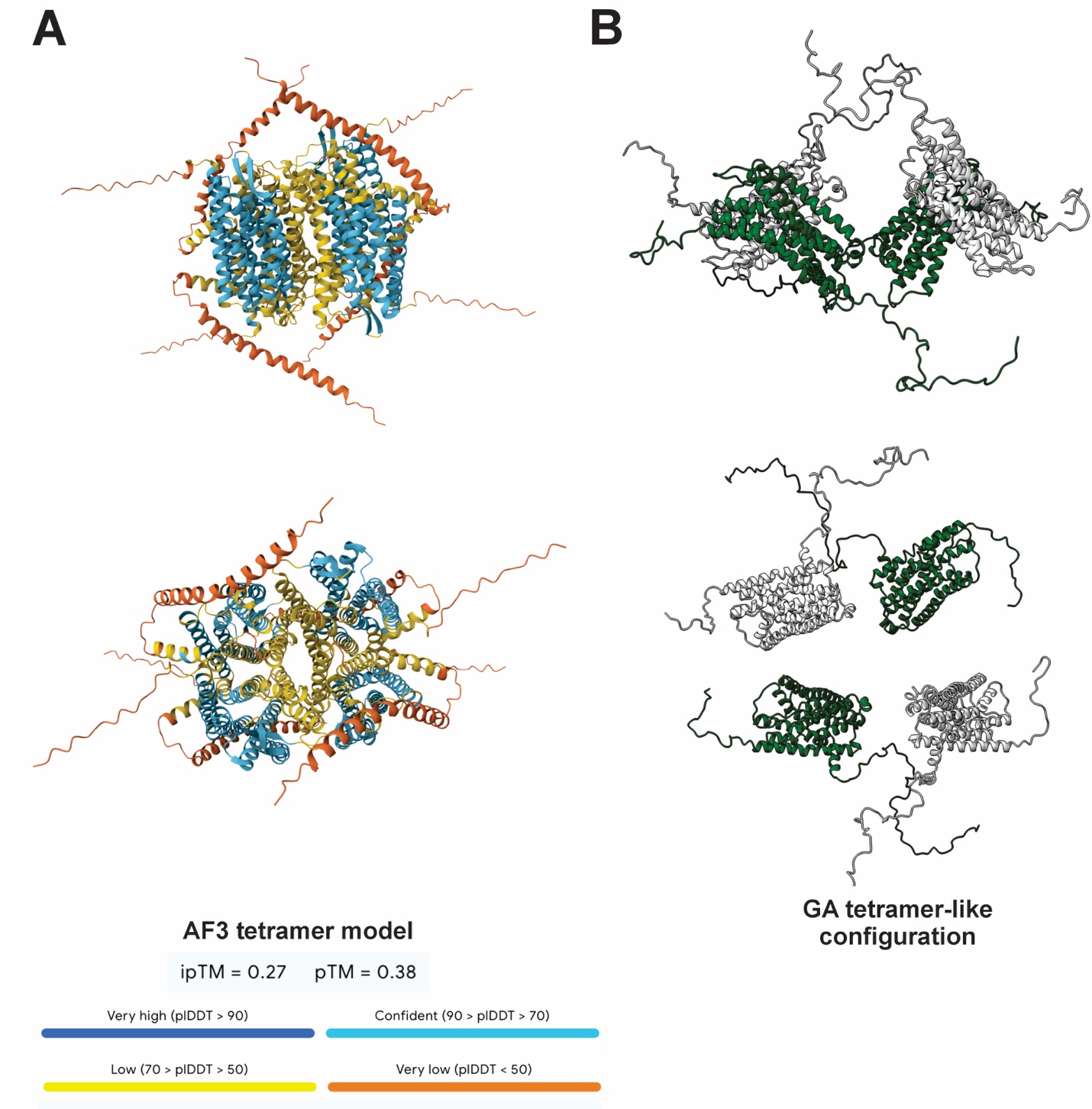


**Figure S7. AlphaFold3-predicted and GA-derived wsMOR tetramer model used for SANS fitting.** *(A*) AF3-predicted wsMOR tetramer, colored by per-residue confidence (pLDDT; scale shown at bottom). (*B*) GA-derived tetramer-like configuration model of wsMOR in solution. AF3-predicted models served as initial structural templates for GA-based simulations. Disordered regions correspond to the N-terminal His and FLAG tags and the C-terminal tails. Some protomers within the homotetramer are colored distinctly for visual clarity.


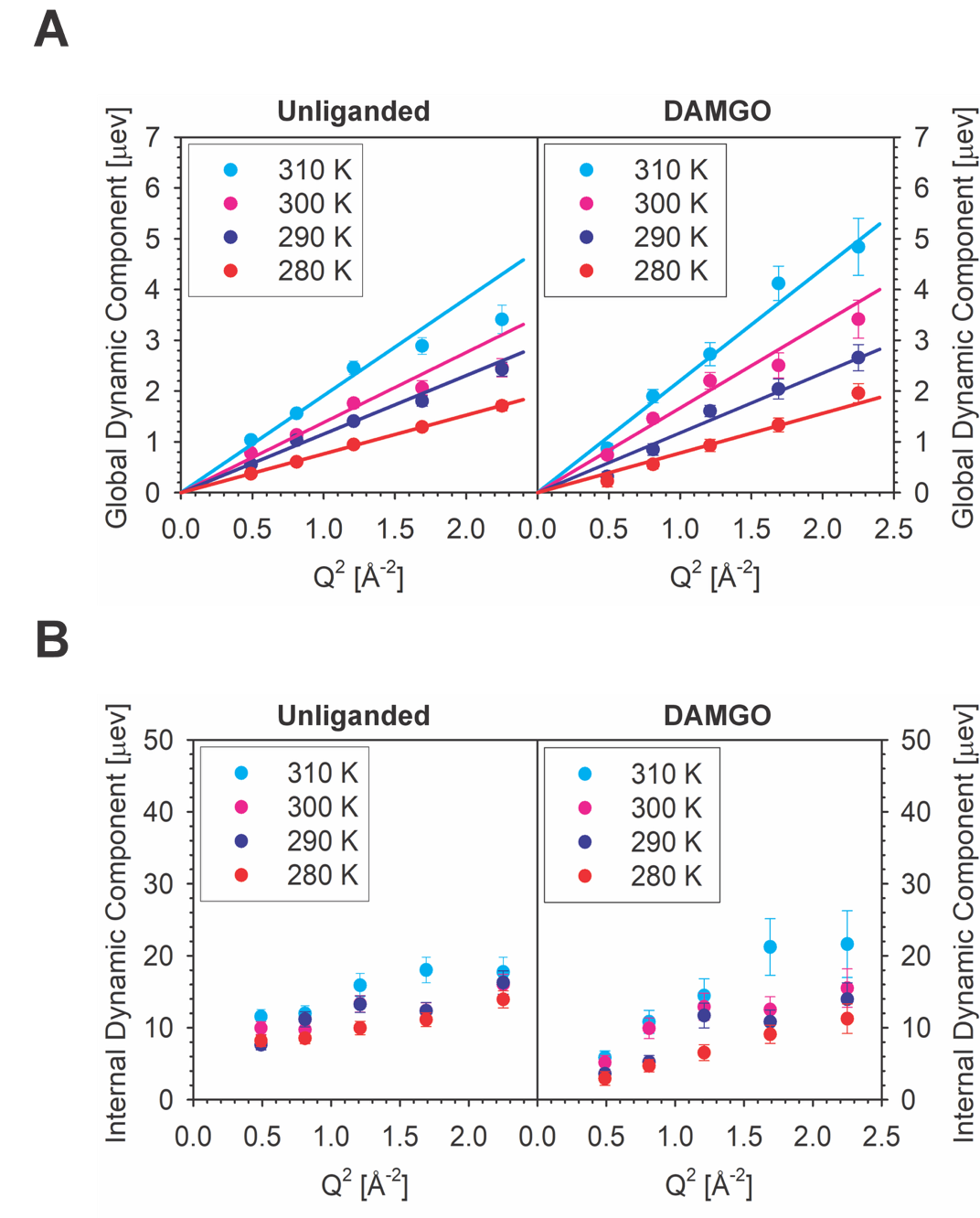


**Figure S8. QENS signal broadening as a function of Q.** *(A)* Narrower (slower) dynamic component fitted with HWHM=DQ^2^, where D is the global diffusivity. *(B)* Broader (faster) dynamic component fitted with HWHM = (1/t_0_)Q^a^, where (1/t_0_) is the inverse relaxation time (the rate) of the internal dynamics.


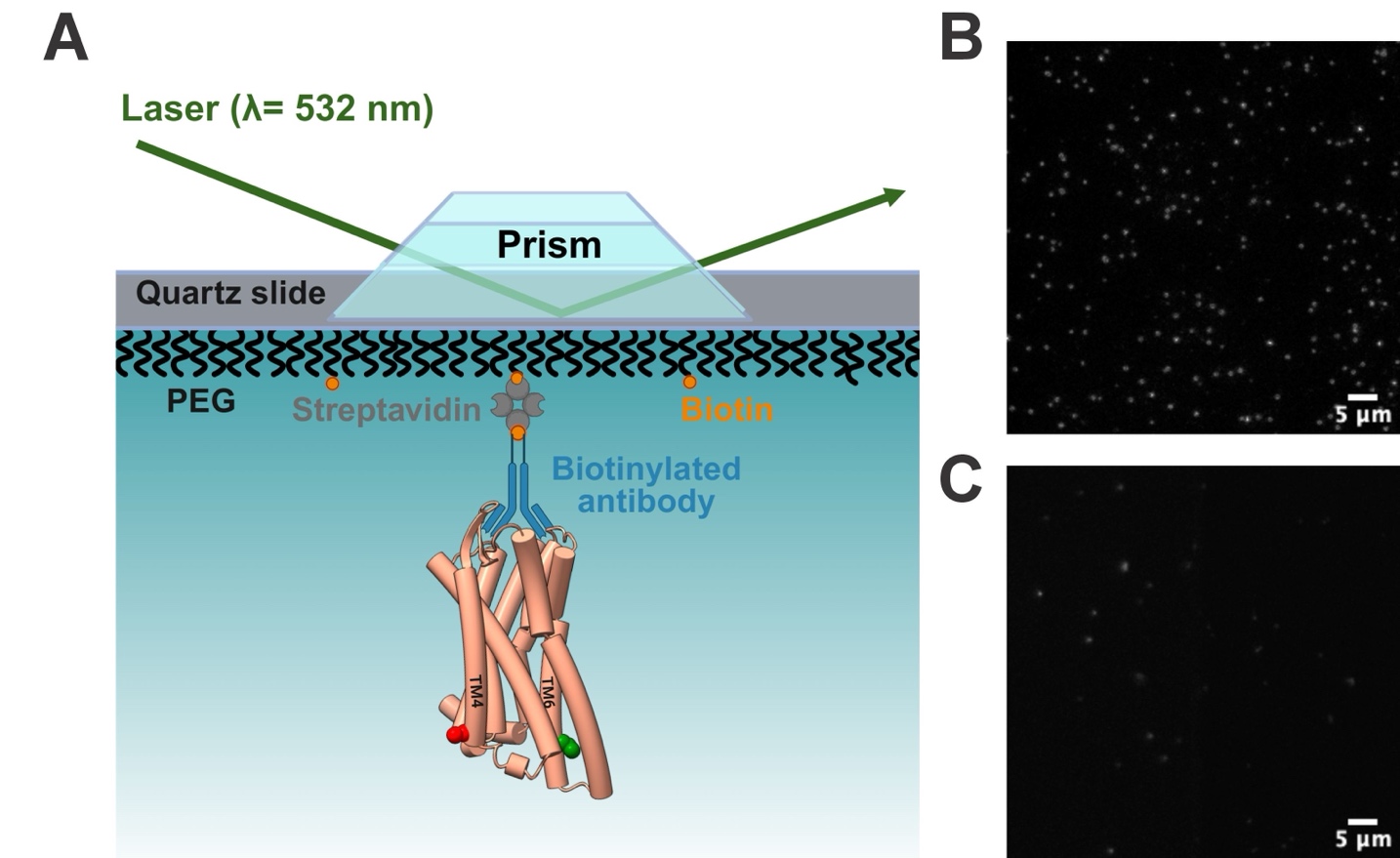


**Figure S9. Single-molecule fluorescence detection of wsMOR.** *(A)* Schematic illustration of wsMOR immobilized on a PEG passivated microscope slide using biotin-streptavidin interactions. Fluorophore labeling sites on helices 4 and 6 are represented as red and green spheres, respectively. *(B)* TIRF microscopy image of immobilized wsMOR labeled with LD555-MAL and LD655-MAL, showing individual receptor spots. *(C)* Control TIRF image without streptavidin binding, displaying significantly fewer fluorescent spots compared to *B*.


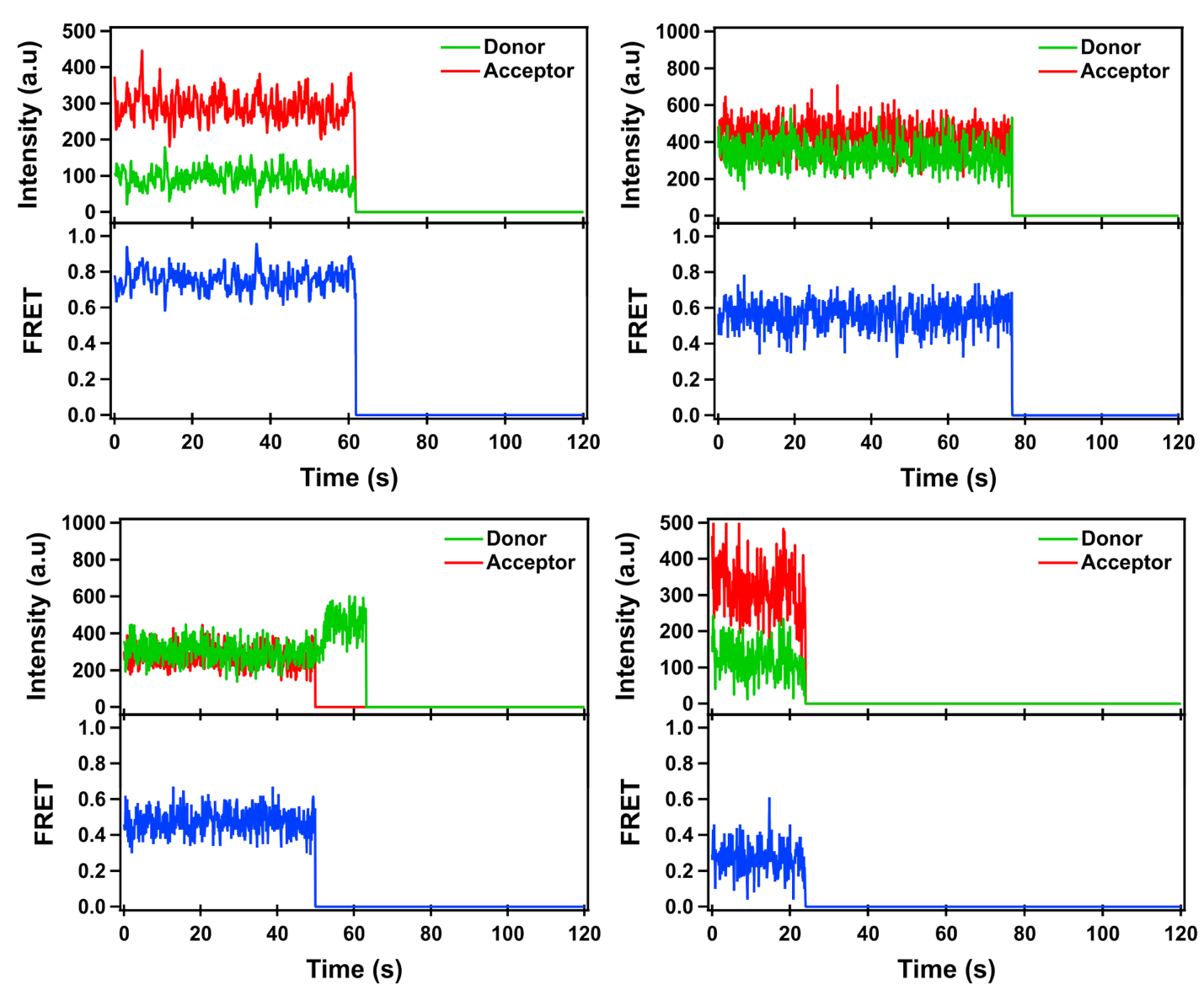


**Figure S10. Single-molecule FRET trajectories of wsMOR.** Each panel shows example fluorescence time trajectories of the four distinct states shown in **Figure 5C-F** (see main text). In each panel, the donor LD555 (green) and acceptor LD655 (red) intensities are depicted at the top, and the FRET efficiency traces, calculated till the photobleaching point, are depicted in blue at the bottom.


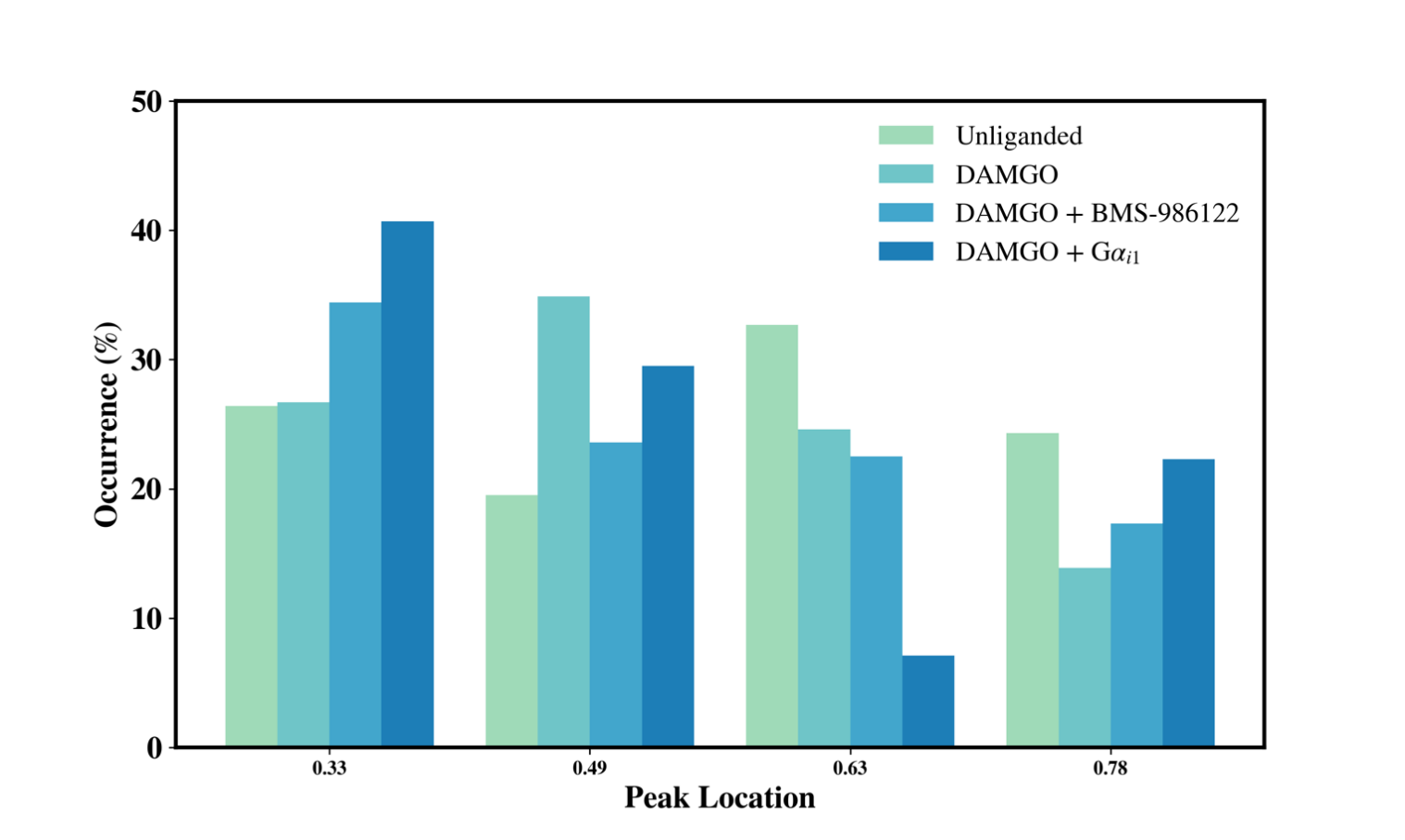


**Figure S11. Relative occurrences of wsMOR TM6 conformational states from smFRET experiments.** The bar chart illustrates the relative population distributions of four distinct FRET efficiency states, corresponding to different TM6 conformations, under various conditions. Each value is shown as an approximate population percentage and represents the area under a FRET peak in the smFRET population histogram, expressed as a fraction of the total area.


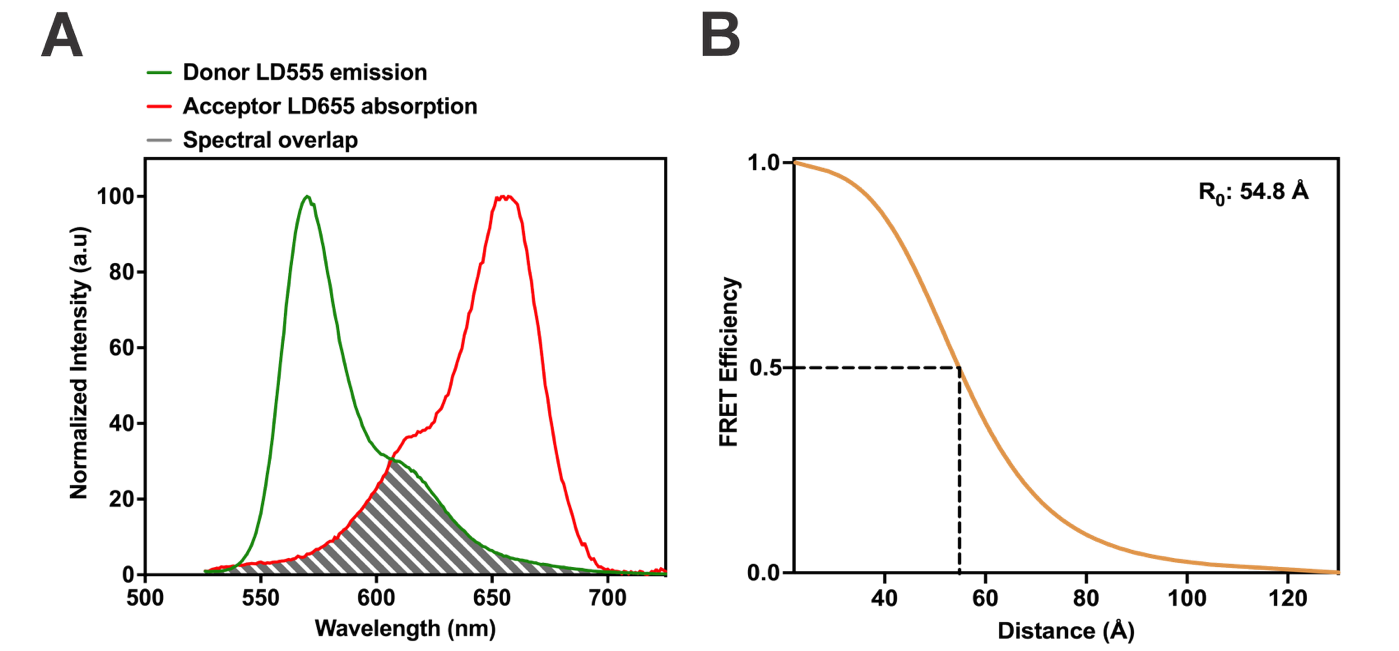


**Figure S12. Förster radius estimation for LD555–LD655 fluorophore pair.** *(A)* Normalized fluorescence emission spectrum of LD555-labeled wsMOR (green) and absorption spectrum of LD655-labeled wsMOR (red), showing the spectral overlap region (gray striped area) of the two fluorophores. Emission was recorded using excitation at 517 nm. *(B)* FRET efficiency as a function of inter-fluorophore distance. The Förster radius (R₀) for the LD555-LD655 pair was calculated to be ~54.8 Å using a donor quantum yield of 0.29 (Lumidyne Technologies), a refractive index of 1.4, an orientation factor (κ²) of 2/3, and a spectral overlap integral *J(λ)* of 6.24×10¹⁵ M⁻¹cm⁻¹nm⁴. The spectral overlap integral was derived from the measured emission spectrum of LD555 and the absorption spectrum of LD655. The extinction coefficient (ε) of LD655 used in the calculation was 250,000 M⁻¹cm⁻¹, as provided by the manufacturer.

Table S1. SAS structural parameters and data fitting.

| **SAS technique**  **employed** | **SAXS** | **SAXS** | **SANS** |
| --- | --- | --- | --- |
| Protein concentration (mg mL^−1^) | 0.7 | 2.7 | 2.5 |
| **Guinier analysis** |  |  |  |
| *I* (0) (cm^−1^) | 1.566 ± 0.045* | 1.508 ± 0.017* | 1.508 ± 0.004 |
| *R*_g_ (Å) | 43.4 ± 0.2 | 59.8 ± 0.9 | 45.4 ± 0.2 |
| *Q*x*R*_g_ range | 0.35-1.28 | 0.48 – 1.25 | 0.43 – 1.18 |
| ***P*(r) analysis** |  |  |  |
| *I* (0) (cm^−1^) | 1.548 ± 0.037* | 1.537 ± 0.014* | 1.525 ± 0.005 |
| *R*_g_ (Å) | 47.3 ± 1.3 | 63.0 ± 0.7 | 47.7 ± 0.3 |
| *D*_max_ (Å) | 153 ± 5 | 244 ± 6 | 180 ± 4 |
| *Q* range (Å^−1^) | 0.008–0.18 | 0.008–0.6 | 0.008–0.22 |
| *χ*^2^ | <1 | 1.31 | 1.02 |
| **Molecular weight (***M* **_W_**) **analysis** |  |  |  |
| Theoretical *M*_W_ (kDa) of monomer |  |  | 42.94 |
| *M*_W_ (kDa) using I(0) | - | - | 48.1 |

*SAXS data is not scaled to an absolute scale

Table S2. Summary of the analysis of analytical sedimentation data for wsMOR.

| **wsMOR** | **Molecular Species** | **Theoretical Mass (KDa)** | **Apparent Mass (kDa)** | **Percentage (%)** | **S_20, w_ (S)**^†^ | **Fit r.m.s.d** | **Frictional ratio** |
| --- | --- | --- | --- | --- | --- | --- | --- |
| 0.5 mg/mL | Monomer  Dimer  Trimer  Tetramer | 42.9  85.8  128.7  171.6 | 52±4  49±1^*^  96±10  74±1^*^  138±13  116±1^*^  158±5  175±1^*^ | 66.9  64.5^*^  13.0  12.5^*^  14.9  15.6^*^  2.0  5.8^*^ | 2.93±0.16  2.86±0.16^*^  4.42±0.16  3.78±0.16^*^  5.63±0.22  5.12±0.16^*^  6.27±0.11 6.75±0.16^*^ | 9.6x10^-3^  8.4x10^-3*^ | 1.4 |
| 2.5 mg/mL | Monomer  Dimer  Trimer  Tetramer | 42.9  85.8  128.7  171.6 | 51±2  90±4  121±6  162±6 | 67.5  7.6  10.1  3.6 | 2.69±0.01  3.94±0.10  4.78±0.18  5.82±0.18 | 0.0288 | 1.8 |
| 10 mg/mL | Monomer  Dimer  Trimer  Tetramer | 42.9  85.8  128.7  171.6 | 55±6  89±8  127±8  198±22 | 41.9  11.9  39.3  3.6 | 2.6±0.15  3.7±0.21  4.6±0.25  6.2±0.29 | 0.0929 | 1.9 |

The molecular mass of the species was estimated based on the theoretical molecular mass of the protein sequence for the monomer, dimer, trimer, and tetramer.

*Data collected using UV absorbance at 280 nm.

^†^The sedimentation coefficient (S_20, w_) was corrected to standard conditions (S_20, w_) at 20 °C.

Unless stated otherwise, data were collected in interference mode.

We estimated the partial specific volumes of the protein and SDS detergent to be 0.7311 mL/g (SEDNTERP) and 0.815 mL/g (1), respectively. Based on mass concentration ratios of SDS to protein, we calculated the weight-average partial specific volumes at varying protein concentrations (0.5, 2.5, and 10 mg/mL), with SDS fixed at 0.01%, to reflect the relative contribution of each component. The resulting values were 0.74508 mL/g at 0.5 mg/mL, 0.7343 mL/g at 2.5 mg/mL, and 0.73197 mL/g at 10 mg/mL. Buffer density and viscosity were estimated using SEDNTERP to be 1.0274 g/mL and 0.01225 Poise, respectively.

Reported errors are the standard deviation of the integrated sedimentation coefficient distribution.

Table S3. Summary of smFRET efficiencies and distances calculated.

| **Condition** | **Cα-Cα** **distance**  **(Crystal/Cryo-EM Structures)** | **TM6 Displacement relative to TM4**  **(Crystal/Cryo-EM Structures)** | **Inter-fluorophore distance**  **(FRET Sensor)** | **TM6 Displacement relative to TM4**  **(FRET Sensor)** |
| --- | --- | --- | --- | --- |
| Inactive | ~ 23 Å  (4DKL) | - | 44.4 Å | - |
| Intermediate-inactive | N/A | N/A | 49.4 Å | ~ 5 Å |
| Intermediate-active | N/A | N/A | 55.2 Å | ~ 10.8 Å |
| Active | ~ 33 Å  (5C1M, 6DDF, and 8EFQ) | ~10 Å  (5C1M, 6DDF, and 8EFQ) | 61.7 Å | ~ 17.3 Å |

**smFRET measurements and distance calculations for wsMOR.** Distance estimates based on structural models of MOR (PDB: 4DKL, 5C1M, 6DDF, and 8EFQ) were calculated using α-carbon distances between the labeling positions on TM4 (R184C) and TM6 (R278C). FRET-derived distances were obtained from experimental measurements using a Förster radius of 54.8 Å and reflect fluorophore-to-fluorophore separations under different conformational states. The high-FRET (inactive) unliganded state (FRET efficiency = 0.78; 44.4 Å) was used as the reference conformation. TM6 displacement was calculated as the increase in inter-fluorophore distance between the stationary TM4 and flexible TM6. Distances derived from X-ray or cryo-EM structures serve as reference points and do not account for fluorophore mobility or linker lengths.

Supplementary References

1. Tanford, C., Nozaki, Y., Reynolds, J. A., and Makino, S. (1974) Molecular characterization of proteins in detergent solutions. *Biochemistry* **13**, 2369-2376
